# Quantitative phase imaging to study transmembrane water fluxes regulated by CFTR and AQP3 in living human airway epithelial CFBE cells and CHO cells

**DOI:** 10.1371/journal.pone.0233439

**Published:** 2020-05-29

**Authors:** Jodie Llinares, Anne Cantereau, Lionel Froux, Frédéric Becq

**Affiliations:** Laboratoire Signalisation et Transports Ioniques Membranaires, Université de Poitiers, Poitiers, France; Emory University School of Medicine, UNITED STATES

## Abstract

In epithelial cells, the cystic fibrosis transmembrane conductance regulator (CFTR), a cAMP-regulated Cl^-^ channel, plays a key role in water and electrolytes secretion. A dysfunctional CFTR leads to the dehydration of the external environment of the cells and to the production of viscous mucus in the airways of cystic fibrosis patients. Here, we applied the quadriwave lateral shearing interferometry (QWLSI), a quantitative phase imaging technique based on the measurement of the light wave shift when passing through a living sample, to study water transport regulation in human airway epithelial CFBE and CHO cells expressing wild-type, G551D- and F508del-CFTR. We were able to detect phase variations during osmotic challenges and confirmed that cellular volume changes reflecting water fluxes can be detected with QWLSI. Forskolin stimulation activated a phase increase in all CFBE and CHO cell types. This phase variation was due to cellular volume decrease and intracellular refractive index increase and was completely blocked by mercury, suggesting an activation of a cAMP-dependent water efflux mediated by an endogenous aquaporin (AQP). AQP3 mRNAs, not AQP1, AQP4 and AQP5 mRNAs, were detected by RT-PCR in CFBE cells. Readdressing the F508del-CFTR protein to the cell surface with VX-809 increased the detected water efflux in CHO but not in CFBE cells. However, VX-770, a potentiator of CFTR function, failed to further increase the water flux in either G551D-CFTR or VX-809-corrected F508del-CFTR expressing cells. Our results show that QWLSI could be a suitable technique to study water transport in living cells. We identified a CFTR and cAMP-dependent, mercury-sensitive water transport in airway epithelial and CHO cells that might be due to AQP3. This water transport appears to be affected when CFTR is mutated and independent of the chloride channel function of CFTR.

## Introduction

Cystic fibrosis (CF), a genetic disease caused by mutations in the gene coding for the epithelial chloride channel CFTR (Cystic Fibrosis Transmembrane conductance Regulator) is characterized by a disruption of the functions of the respiratory system, digestive tract and reproductive tract [1]. In the airways, the absence of CFTR at the plasma membrane or a change in its function induces dehydration of the surface fluid and the production of abnormally thick mucus [2]. Protection against pathogenic microorganisms contained in inhaled air is then impaired, which can lead to inflammation and lung infections, the first cause of morbidity in CF patients [3].

The CFTR channel is a protein belonging to the ABC (ATP Binding Cassette) transporter family [4] which is composed of two MSD (Membrane Spanning Domain) and two NBD (Nucleotide Binding Domain) domains, NBD1 and NBD2, containing ATP binding sites. The two MSD-NBD tandems are linked by a regulatory domain R [5]. Activation of cAMP-dependent kinases (PKA and PKC) that phosphorylate the R domain and hydrolysis of the ATP fixed on the NBD domain induce a change in channel conformation leading to its opening [6–9]. The F508del mutation, corresponding to the deletion of a phenylalanine in position 508, is the most common mutation found in CF patients (90% of them possess at least one CFTR allele bearing the F508del mutation). F508del-CFTR mutation is characterized by a lack of protein maturation (misfolding) resulting in its retention in the endoplasmic reticulum, early degradation by the ubiquitin-proteasome machinery, reduced residence time at the plasma membrane and the almost complete absence of expression at the apical membrane [10,11]. Even if a small amount of protein can reach the apical membrane, the channel has an altered gating with longer closing episodes than its wild-type counterpart [12]. Small synthetic molecules, called correctors, restore in some conditions the expression of the F508del-CFTR protein to the membrane surface by correcting its folding defect [13]. This is the case of the corrector VX-809, developed by Vertex Pharmaceuticals Inc. and marketed as Lumacaftor [14]. Other molecules, named potentiators, increase the open probability of the CFTR channel following its R-domain phosphorylation. The most effective one being VX-770 (Ivacaftor) that increases G551D- and F508del-CFTR channels activity independently of ATP hydrolysis [15].

In the lungs, the hydration of airway surfaces is critically dependent on water and electrolytes transepithelial absorption and secretion. At the apical plasma membrane, the epithelial sodium channel (ENaC) is responsible for sodium absorption whereas the chloride secretion occurs by CFTR and TMEM16A, a Ca^2+^ activated Cl- channel. These two ion channels are involved in Airway Surface Liquid (ASL) regulation which is necessary to mucus clearance and to the maintenance of lung sterility (reviewed in [2]). Epithelial water transport is osmotically driven by ionic fluxes and mainly occurs through two pathways: the transcellular pathway through aquaporins and the paracellular pathway (between the cells) [16]. Because mutations in the CFTR gene disrupt chloride transport at the apical membrane of epithelial cells and lead to perturbed water secretion, it is important to develop methods to study water movement related to the function of CFTR. Indeed, airway surface hydration might constitute a therapy to various pulmonary diseases like CF and chronic obstructive pulmonary disease (COPD) [17,18]. During the past two decades, a novel method to investigate transmembrane water transport regulation in living cells has been developed. It is based on the Digital Holographic Microscopy (DHM), a non-invasive and label-free quantitative phase imaging technique detecting light retardation induced by transparent specimen called phase shift [19–24]. The measured phase signal is dependent on the intracellular and extracellular refractive indexes and the cellular thickness. In particular, the authors concluded that phase signal variations are more sensitive to intracellular refractive index changes (through dilution or concentration of intracellular medium) than cell volume variations during transmembrane water movements [20]. A phase decrease was then observed during water influx whereas a phase increase was detected during a water efflux. In particular, the study of Jourdain et al. showed that CFTR can activate an AQP3-mediated and cAMP-dependent water efflux in CHO cells [22]. Among other quantitative phase imaging techniques, a module based on quadriwave lateral shearing interferometry (Phasics© wave-front sensor) was first adapted by Bon et al. to study membrane dynamics and to visualize intracellular components [25]. Thereafter, it was also used to measure quantitative information such as cellular dry mass (total cellular mass without water) [26]. Unlike DHM, this system is compatible with a white-light illumination microscope. Moreover, it samples the phase in the wave front sensor plane and therefore does not need a reference arm.

In this study, we then adapted the quadriwave lateral shearing interferometry (QWLSI) to record OPD variations in response to water fluxes. The aim was to investigate the role of CFTR channel in transmembrane water transport in human airway epithelial CFBE expressing wild-type (WT) CFTR or F508del-CFTR and in CHO cells expressing wild-type (WT) CFTR, G551D- or F508del-CFTR. Our results highlighted the role of CFTR in a cAMP-dependent and mercury-sensitive water transport in airway epithelial and CHO cells.

## Material and methods

### Cell culture

All cell lines were cultured at 37°C in 5% CO_2_. CFBE41o- cells, provided by Dr. D. Gruenert (Univ. California San Francisco, USA), overexpressing wild-type CFTR (CFBE WT-CFTR) or F508del-CFTR (CFBE F508del) were grown in Eagle’s Minimum Essential Medium (EMEM) with non-essential amino acids (NEAA) (Gibco 10370), 10% fetal bovine serum (FBS) (Sigma), 2 mM L-glutamine (Gibco), 50 IU/ml of penicillin (Sigma) and 50 μg/ml of streptomycin (Sigma)_._ Cells selection was done with 5 μg/ml of puromycin (Gibco). CHO cells (CHO K1) stably expressing wild-type CFTR (CHO WT-CFTR), F508del-CFTR (CHO F508del) and G551D-CFTR (CHO G551D) were grown in alpha minimal essential medium (Gibco 32561) with 7% fetal bovine serum (Sigma), 50 IU/ml of penicillin (Sigma) and 50 μg/ml of streptomycin (Sigma). Selection for CHO WT-CFTR, CHO F508del and CHO G551D cells was done by adding 100 μM, 200 μM and 20 μM of methotrexate (Sigma) in culture medium, respectively. Functionally detectable levels of F508del-CFTR were restored to the plasma membrane by incubating cells with VX-809 (3 μM) for 24h prior experiments. Control treatment was realized with DMSO (0.1% v/v).

### Quantitative phase imaging

**Fig 1:**
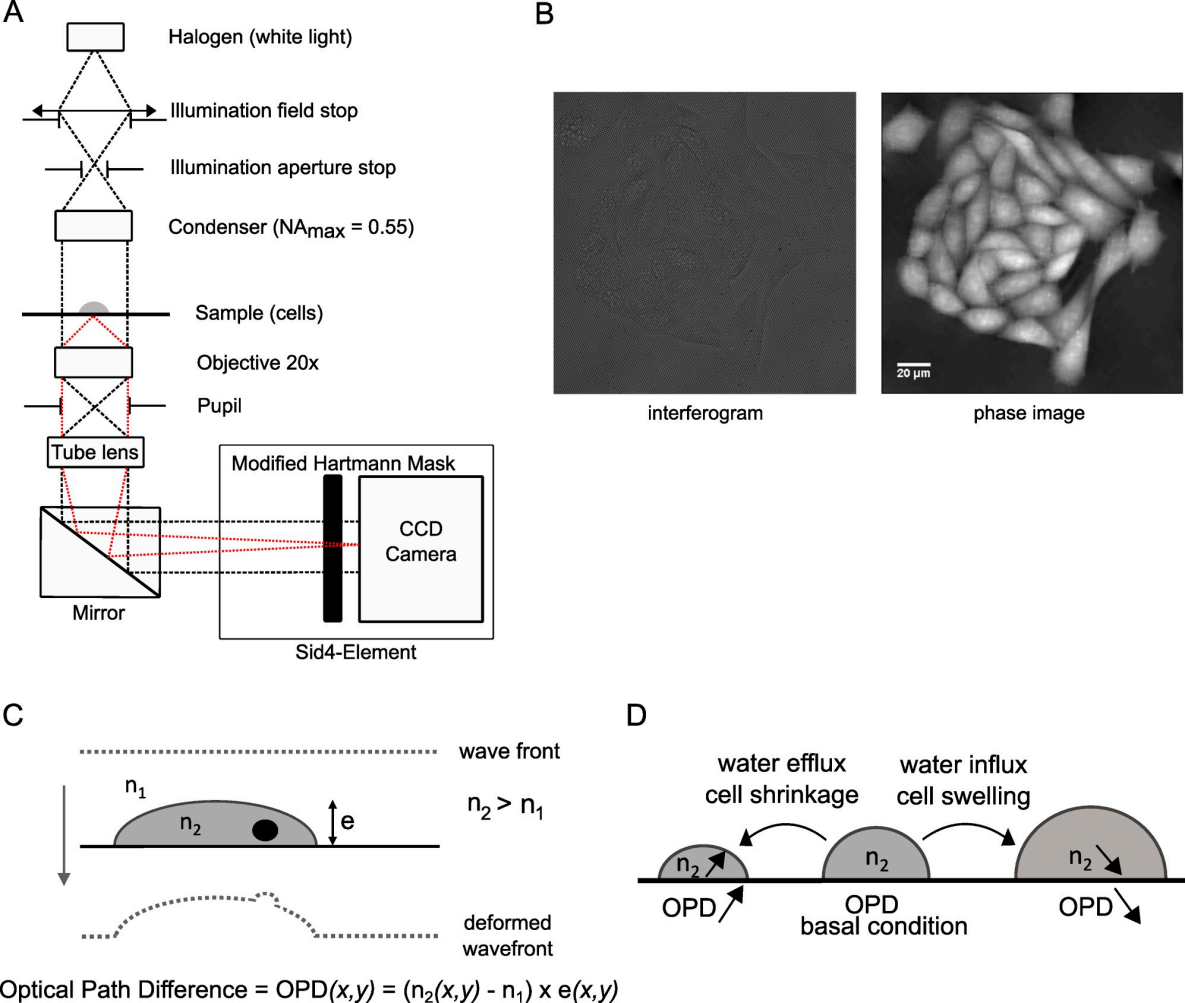
Quantitative phase imaging experimental setup and principle. (A) Schematic representation of the experimental setup composed by a Sid4-Element component (PHASICS, Saint-Aubin, France) connected to a conventional microscope with a halogen source (adapted from [25]). Black lines: illumination path; red lines: objective light detection path. (B) Representative images of a recorded interferogram (left) and its corresponding phase image (right) obtained with the Sid4Bio Software (PHASICS, Saint-Aubin, France). (C) Schematic representation of a light wave front deformation when passing through a cellular sample that is converted in Optical Path Difference (OPD) values by the Sid4Bio system. n_1_: extracellular refractive index, n_2_: intracellular refractive index, e: cell thickness. (D) Schematic representation of Optical Path Difference variation linked to net transmembrane water fluxes. A water efflux leads to an OPD increase corresponding to an increase of n_2_ whereas a water influx leads to an OPD decrease corresponding to a decrease in n_2_.

The Sid4Bio system (PHASICS, Saint-Aubin, France) was used to acquire quantitative phase images by quadriwave lateral shearing interferometry (QWLSI). This setup is composed of a wave front sensor (SID4-Element) inserted between an inverted microscope (IX81, Olympus) and an Andor Zyla5.5 sCMOS camera (Andor, France) (Fig 1A). Halogen white-light source was used for trans-illumination of the sample through a Köhler condenser (NA = 0.55). Images were acquired with x20 objective lens (NA = 0.7). The SID4-Element is composed by a 2D diffraction grating corresponding to a Modified Hartmann Mask (MHM), which captures the input light wavefront and produces four propagating replicas that are interfering to form an interferogram. This image is then captured by the camera and analyzed with a specific algorithm (Fourier integration) by the SiD4Bio software (Fig 1B). For each acquisition, a reference image was obtained in a cell free region to remove OPD variations induced by the microscope components or the illumination. Final phase images were used to measure light deformation when passing through cells, corresponding to an optical path difference (OPD). The OPD is expressed in nanometers and is related to the cell and medium refractive indexes and to cell thickness through the following equation:

$$\mathrm{OPD}\left(x,y\right)=\left(n_{2}\left(x,y\right)-n_{1}\right)\times e\left(x,y\right)$$

where n_1_ is the extracellular medium refractive index, n_2_(x,y) is the mean intracellular refractive index at the (x,y) position (with n_2_ > n_1_) and e(x,y) is the cell thickness (Fig 1C).

The Optical Volume Difference (OVD), which is linked to cell dry mass (total cell mass without water), can be measured by integrating the OPD over the entire cell area and is given in μm^3^ [26].

### Phase images acquisition

Cells were grown to 60% confluence on 30 mm diameter #1 ½ glass coverslips (coated with fibronectin for CFBE cells). For phase images acquisition at room temperature, a coverslip was mounted on a POCmini chamber system in 2 ml of extracellular bath solution containing (in mM): 150 NaCl, 3 KCl, 5 D-glucose, 10 HEPES, 3 CaCl_2_, 3 MgCl_2_, pH 7.4 (titrated with NaOH) (325 ± 5 mOsmol). The refractive index of the solution measured with an Abbe refractometer (Wincom Company Ltd) was 1.3342. Culture medium was replaced by the bath solution 10 min before the experiment to equilibrate cell membrane ion transports. Interferograms were acquired during 500ms (exposure time of the camera). Average of 20 images allowed to improve signal to noise ratio. Stacks of reconstructed phase images were then obtained with an interval of 10 s during 20 or 30 min. With this magnification, images contained 20 to 50 cells. Before any stimulation, OPD baseline was recorded for 5 min. For osmotic challenges, addition of ultra-pure water to reach an osmolarity of 165 mOsmol (hypoosmotic medium) whereas NaCl was added to the extracellular medium to reach an osmolarity of 525 mOsmol (hyperosmotic medium). This resulted in refractive indexes alterations from the extracellular bath solution of -0.0007 and 0.0013, respectively.

### Phase images analysis

After acquisition, phase images were analyzed with ImageJ software (NIH). Regions of interest were defined in the center of each cell (see S1 Fig) and over the whole cell surface to measure the OPD and the OVD, respectively. Values before the stimulation (OPD_1_ or OVD_1_, 5 min recording) and values after the stimulation (OPD_2_ of OVD_2_, after 30 min recording except for HgCl_2_ experiments) were measured for each cell. Variations in OPD or OVD were then obtained by subtracting OPD_1_ to OPD_2_ and OVD_1_ to OVD_2_, respectively. Finally, mean OPD or OVD variation of all cells within the field was calculated (one image corresponds to one field per dish). Thus, n field corresponds to n experiments except for the decoupling procedure experiment were n represents the number of cells. Data were analyzed with the GraphPad Prism 5.0 software. Results are expressed as mean ± SEM except for scattered plots representations with the median and upper and lower quartile or box plots representations with the median, boxes as upper and lower quartile and whiskers as minimum and maximum value. Statistical comparisons were made using a two-tailed Mann-Whitney test for unpaired data or a Wilcoxon signed rank test for paired data (* p < 0.05, ** p < 0.01, *** p < 0.001, ns: non significant) with a significance threshold set at p < 0.05.

### Decoupling procedure principle

The decoupling procedure experiment based on the work of Rappaz et al. [20] allows to retrieve mean cellular thickness and mean intracellular refractive index separately from OPD values. We used Nycodenz (Histodenz™, C19H26I3N3O9, MW 821.14) a hydrophilic molecule used in equal molarity to mannitol to shift the refractive index [20]. Two iso-osmotic solutions were prepared by replacing 25 mM of NaCl of the extracellular bath solution by 50 mM of Mannitol or 50 mM of Nycodenz. The refractive indexes of the mannitol and Nycodenz solution were 1.3355 (n_1_) and 1.3405 (n_1_+Δn_1_) respectively. Phase imaging acquisition was first performed with the mannitol solution which was then replaced by the Nycodenz solution. It allowed to measure a first OPD signal with the mannitol solution corresponding to:

$${\mathrm{OPD}}_{1}(x,y)=(n_{2}(x,y)-n_{1})\times e(x,y)$$

and a second OPD signal with the Nycodenz solution corresponding to:

$${\mathrm{OPD}}_{2}(x,y)=(n_{2}(x,y)-(n_{1}+\Delta n_{1})\times e(x,y)$$

The equation system can be solved to obtain the intracellular refractive index:

$$n_{2}(x,y)=\frac{\Delta n_{1}\times {\mathrm{OPD}}_{1}(x,y)}{{\mathrm{OPD}}_{1}(x,y)-{\mathrm{OPD}}_{2}(x,y)}+n_{1}$$

and the cellular thickness:

$$e(x,y)=\frac{{\mathrm{OPD}}_{1}(x,y)-{\mathrm{OPD}}_{2}(x,y)}{\Delta n_{1}}$$

Mean intracellular refractive index $\bar{n_{2}}$ and mean cellular thickness $\bar{e}$ were obtained by averaging OPD_1_ and OPD_2_ values over the whole cell surface. The absolute cell volume was then estimated from the following equation:

$$V_{c}=\bar{e}\times S_{c}≅\bar{e}\times A_{p}\times N_{c}$$

where S_c_ is the projected surface of the cell, A_p_ is the area of a magnified pixel and N_c_ is the number of pixels corresponding to the cell surface.

### RNA extraction and RT-PCR

For reverse-transcript polymerase chain reaction (RT-PCR) experiments, total RNA was extracted from cells using RNable (Eurobio, France). RNA quality and quantity were assessed with a Nanodrop spectrometer (Nanodrop). Reverse transcription of RNA to cDNA was achieved using 40 U Recombinant Ribonuclease Inhibitor (RNaseOUT™, Invitrogen) and 200 U Reverse Transcriptase (Moloney Murine Leukemia Virus Reverse Transcriptase, Invitrogen). The final PCR reaction volume corresponded to 50 μl and contained 5 μl 10X PCR buffer (200 mM Tris-HCl pH 8.4, 500 mM KCl), 4 μl MgCl_2_ buffer (25 mM), 0.5 μl dNTP (25 mM), 0.5 μl forward and reverse primers (20 mM), 180 ng cDNA and 0.5 μl *Taq* DNA Polymerase. The PCR reaction was performed in a Thermocycler under the following conditions: 2 cycles of 5 min at 94°C and 2 min at 60°C then 40 cycles of 30 s at 72°C, 30 s at 94°C and 30 s at 60°C followed by a final elongation step of 5 min at 72°C. The sequence primers (see S1 Table) were designed using the National Center for Biotechnology Information (NCBI) sources. GAPDH was used as house-keeping gene control. RT-PCR products were visualized on 2% agarose gel containing 0.01% Ethidium Bromide.

### Patch-clamp recordings

Voltage clamp recordings in whole cell configuration were performed at room temperature with an Axopatch 200B amplifier connected to a Digidata 1440A interface. Cells were perfused in an external bath solution containing (in mM): 145 NaCl, 4 CsCl, 1 CaCl_2_, 1 MgCl_2_, 10 D-glucose, pH 7.4 (titrated with NaOH) (315 ± 5 mOsmol). Pipettes were pulled from borosilicate glass capillaries (GC150TF-10, Harvard Apparatus) and had a resistance of 4–6 MΩ when filled with an internal solution containing (in mM): 113 L-aspartic acid, 113 CsOH, 27 CsCl, 1 NaCl, 1 MgCl_2_, 1 EGTA, 10 TES and 3 Mg-ATP, pH 7.2 (titrated with CsOH) (285 ± 5 mOsmol). Pipette capacitance was electronically compensated after cell-attached configuration achievement. Membrane potential was held at -40 mV and currents were elicited from -100 mV to +100 mV by 20 mV step increments. All drugs were added to the external bath solution and perfused with a gravity perfusion system. The majority of non CFTR-dependent chloride conductances were blocked by adding 4,4’-diisothiocyanatostilbene-2,2’-disulfonic acid (DIDS, 200 μM) to the bath solution. Data were analyzed with pCLAMP 9.0 software (Molecular Devices) and GraphPad Prism 5.0 software. Results are expressed as mean ± SEM, with n corresponding to the number of cells, or are represented by box plots with the median, boxes as upper and lower quartile and whiskers as minimum and maximum value. Statistical comparisons were performed using a two-tailed Mann-Whitney test (* p < 0.05, ** p < 0.01, *** p < 0.001, ns: non significant) with a significance threshold set at p < 0.05.

### Chemical reagents

Forskolin, mercuric chloride (HgCl_2_), CFTR(inh)-172, DIDS (4,4’-diisothiocyanatostilbene-2,2’-disulfonic acid), genistein and Nycodenz (Histodenz™) were purchased from Sigma Aldrich (France). VX-770 and VX-809 were obtained from Selleckchem (USA). Stock solutions (1000X) were prepared using dimethyl sulfoxide (DMSO) as solvent.

## Results

### Recording water fluxes in living cells with QWLSI

**Fig 2:**
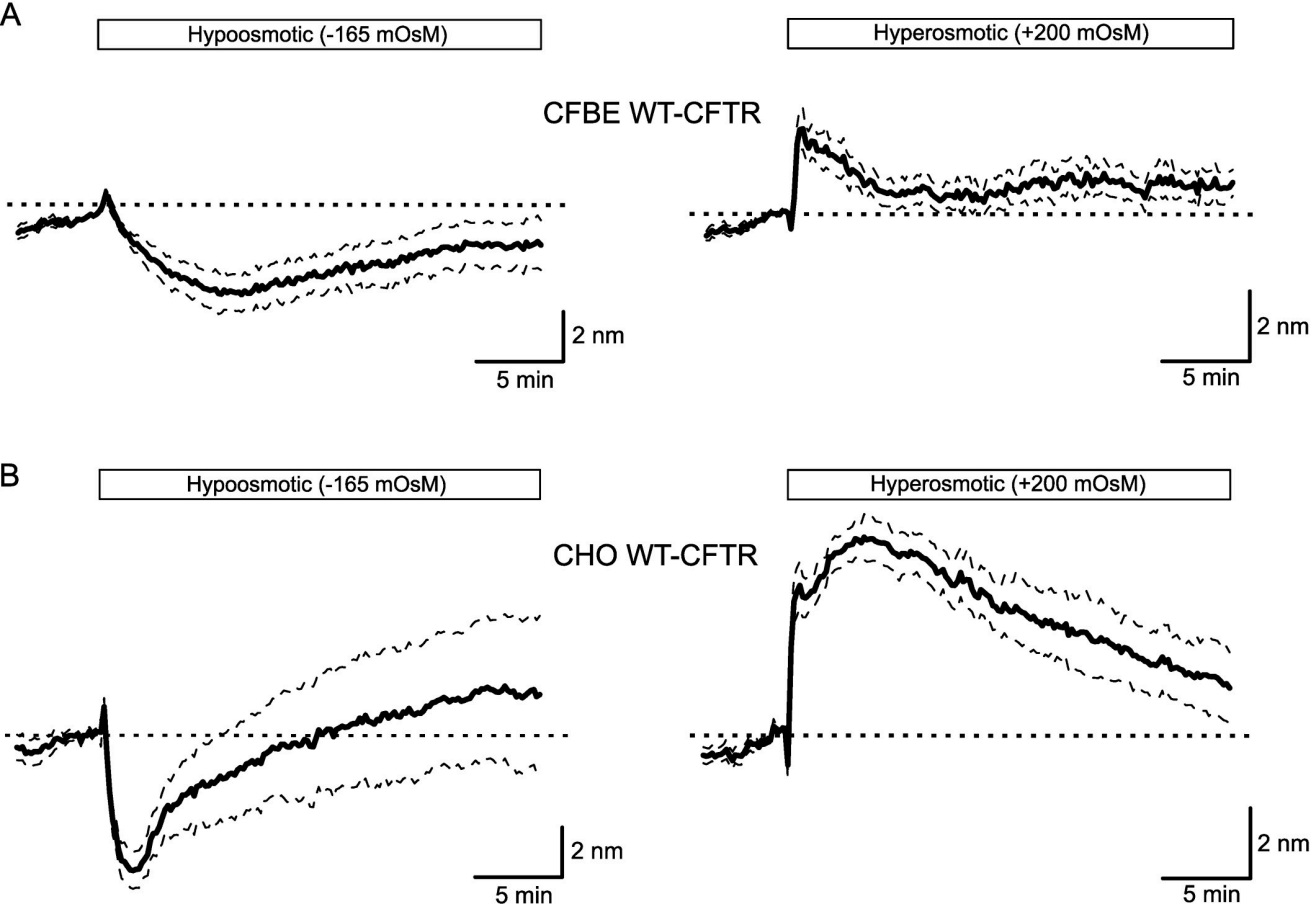
Optical Path Difference (OPD) variations recorded during osmotic stress. (A) Time course of OPD variation measured for CFBE WT-CFTR cells during incubation with hypotonic (left traces, n = 7) and hypertonic (right traces, n = 7) extracellular solutions. (B) Time course of OPD variation measured for CHO WT-CFTR cells during incubation with hypotonic (left traces, n = 8) and hypertonic (right traces, n = 7) extracellular solutions and corresponding schematic representations of water fluxes consequences on the intracellular refractive index n_2_.

To study water transport regulation in living cells, we used the quadriwave lateral shearing interferometry (QWLSI), a quantitative phase imaging technique that allowed us to follow variations of the optical path difference (OPD), a parameter depending on intracellular and extracellular refractive indexes (n_2_ and n_1_, respectively) and cell thickness (e) (Fig 1C, see material and methods). Transmembrane water movements are controlled by changes in intracellular and extracellular osmolarity. A hypotonic stress, corresponding to a decrease in extracellular osmolarity, activates osmotic water influx and cell swelling whereas a hypertonic stress, corresponding to an increase in extracellular osmolarity, activates osmotic water efflux and cell shrinkage (Fig 1D). According to the studies realized with the DHM technique, OPD values are expected to decrease during water influx (hypotonic stress) mainly due to the dilution of intracellular content leading to intracellular refractive index (n_2_) decrease [20,23]. On the contrary, OPD values are expected to increase following water efflux (hypertonic stress) that induces intracellular content concentration and n_2_ increase. In both cases, these changes in cell volume are counteracted by two regulatory mechanisms: the regulatory-volume decrease (RVD) and the regulatory-volume increase (RVI) activated by cell swelling and cell shrinkage, respectively [27–29]. To validate this approach in our cellular models, we first recorded OPD variations during osmotic stress in both CFBE WT-CFTR and CHO WT-CFTR cells. A hypotonic shock of 50% (-165 mOsmol) induced a transient OPD decrease in both CFBE WT-CFTR (Max OPD shift: -3.24 ± 0.70 nm, n = 7; Fig 2A left) and CHO WT-CFTR cells (Max OPD shift: -5.51 ± 0.73 nm, n = 8; Fig 2B left), suggesting an activation of water influx followed by a RVD mechanism. On the opposite, a hypertonic shock (+200 mOsmol) activated a transient OPD increase in CFBE WT-CFTR (Max OPD shift: 2.46 ± 0.59 nm, n = 7; Fig 2A right) and CHO WT-CFTR cells (Max OPD shift: 5.66 ± 0.65 nm, n = 7; Fig 2B right) corresponding to water efflux, followed by an OPD decrease, suggesting the activation of a RVI process. In CHO WT-CFTR cells, both responses to osmotic challenges were larger than CFBE WT-CFTR cells. As illustrated in S1 Fig, showing representative phase images for each condition, cells are remaining adherent and do not undergo drastic morphological changes during hypo- or hypertonic stresses. It is important to consider that in order to cause osmotic stresses, the solutions used here have a modified refractive index (n_1_) compared to the normal extracellular medium (n_1_ = 1.3342). Indeed, adding ultra-pure water to decrease the normal solution osmolarity (hypotonic solution) induces a n_1_ decrease of 0.0007. On the contrary, adding NaCl to increase the normal solution osmolarity (hypertonic solution) induces an n_1_ increase of 0.0013. Analysis of the OPD variation during time course showed very small and transient variations due to these n1 changes that modifies (n2-n1) and induce an initial rise of OPD for the hypoosmotic solution (due to n1 decrease) and, on the contrary, an initial drop of OPD for the hyperosmotic solution (n1 increase). This OPD variation is rapidly reversed by large OPD variation due to the activation of cellular response to osmotic challenges. Based on what has been described at the beginning of this paragraph, these large OPD variations measured during osmotic stresses are most probably resulting from intracellular refractive index modification (n_2_) due to dilution or concentration of intracellular content [20,23]. Therefore, this first set of experiments suggests that OPD variations induced by transmembrane water fluxes following osmotic challenges could be monitored by QWLSI.

### Forskolin induces an OPD increase in CFBE and CHO WT-CFTR cells

**Fig 3:**
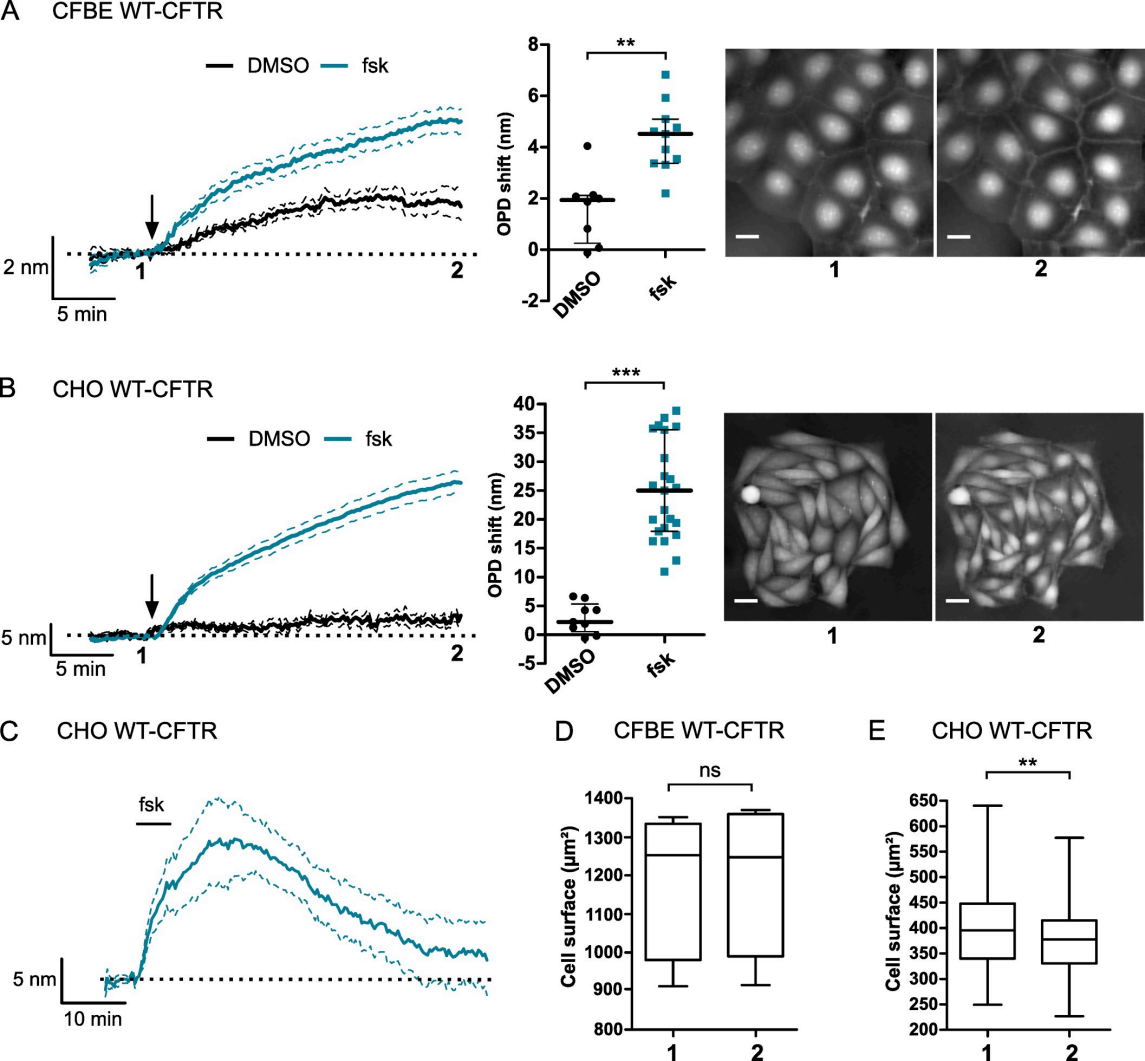
Stimulation of an OPD increase by forskolin application in CFBE and CHO overexpressing the wild-type CFTR channel. (A) Time course (left) and quantification (middle) of OPD variation during forskolin stimulation (fsk, arrow, 10 μM, n = 11) compared to control condition with DMSO (0.1%, n = 8, ** p < 0.01, two-tailed Mann-Whitney test) of CFBE WT-CFTR cells. Representative phase images (right) before (1) and after 25 min of forskolin stimulation (2). (B) Time course (left) and quantification (middle) of OPD variation during forskolin stimulation (fsk, arrow, 10 μM, n = 23) compared to control condition with DMSO (0.1%, n = 9, *** p < 0.001, two-tailed Mann-Whitney test) in CHO WT-CFTR cells. Representative phase images (right) before (1) and after 25 min of forskolin stimulation (2). (C) OPD variation after short forskolin application (5 min) in CHO WT-CFTR cells (n = 5). (D) Cell surface quantification (μm^2^) of CFBE WT-CFTR cells before and after forskolin application (n = 10, p = 0.1934, Wilcoxon signed rank test). (E) Cell surface quantification (μm^2^) of CHO WT-CFTR cells before and after forskolin application (n = 20, p = 0.0263, Wilcoxon signed rank test). Scale bar: 20 μm.

To explore the role of CFTR in water transport regulation, we recorded OPD variation after adding forskolin to the bath solution. Forskolin is an adenylate cyclase activator broadly used to stimulate CFTR activity through an increase in intracellular cAMP concentration. Adding forskolin (10 μM) induced an OPD increase in CFBE WT-CFTR (4.37 ± 0.39 nm, n = 10; Fig 3A) and CHO WT-CFTR cells (24.89 ± 1.78 nm, n = 23; Fig 3B). This OPD shift stabilized around 25 min of incubation and was significantly different from control stimulation (DMSO) in both cell types (p = 0.0015 and p < 0.001, respectively; Fig 3A and 3B). Moreover, this OPD increase was reversed by washing forskolin from the extracellular medium after 5 minutes of stimulation, as illustrated for CHO WT-CFTR cells in Fig 3C (n = 5). These results showed that, in CFBE and CHO cells expressing WT-CFTR, an OPD variation can be activated by increasing intracellular cAMP levels. Our observations are in agreement with the results published by Jourdain et al. using the DHM technique showing that forskolin activated a phase increase in CHO WT-CFTR cells. The authors concluded that this variation was due to an increase of the intracellular refractive index induced by a transmembrane water efflux [22].

However, this OPD increase could also be due to a change in cell morphology, we then measured the surface of CHO and CFBE WT-CFTR cells before and after the forskolin stimulation (10 μM). We did not detect any significant variation of CFBE WT-CFTR cell surface after application of forskolin (basal: 1179 ± 56.84 μm^2^; forskolin: 1186 ± 57.50 μm^2^, n = 10, p = 0.1934; Fig 3D). On the other hand, the same stimulation induced significant decrease of CHO WT-CFTR cell surface by 6.4% (basal: 406.7 ± 21.29 μm^2^, forskolin: 380.5 ± 20.88 μm^2^ (n = 20, p = 0.0263, Fig 3E).

### Variation of cellular biophysical parameters after forskolin application in CHO WT-CFTR cells

**Fig 4:**
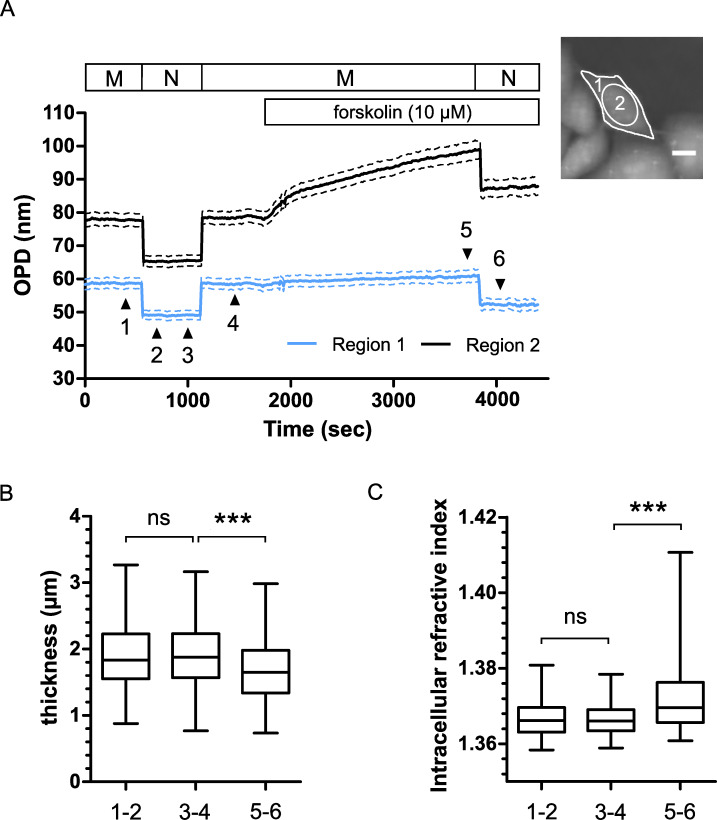
Phase decoupling procedure performed on CHO WT-CFTR cells. (A) Graphical representation of mean OPD variations of CHO WT-CFTR cells (n = 81 cells) recorded over the whole cell surface or in the nucleus region (region 1 and region 2 respectively, represented on the quantitative phase image inserted on the top right, scale bar: 10 μm). The rectangles above the recorded signals represent the sequence of the extracellular solutions used for the decoupling procedure (M: Mannitol solution, N: Nycodenz solution) and the application of forskolin. (B, C) Quantification of mean cellular thickness (B) and mean intracellular refractive index (C) before (1–2, 3–4) and after (5–6) forskolin stimulation obtained from OPD values of region 1. (*** p < 0.001, Wilcoxon signed rank test).

Because the CHO WT-CFTR cell surface reduction described above could be accompanied by a cell thickness increase which could by itself explain the OPD increase, we then performed a decoupling procedure as described by Rappaz et al. [20]. This procedure allows to retrieve two biophysical parameters, the cell thickness and the integral intracellular refractive index, separately from the mean cellular OPD (see material and methods). To do so, we recorded quantitative phase images of CHO WT-CFTR cells with a first physiological solution containing mannitol with a refractive index of n_1_ = 1.3355. This medium was then replaced by a second solution containing an equal molarity of Nycodenz instead of mannitol, to avoid cell volume variations, which increases the refractive index by Δn_1_ = 0.005 and then reduced the OPD signal values. Fig 4A shows the mean OPD signal of the whole cell surface (region 1) or from the nucleus region (region 2) recorded during the decoupling procedure steps performed on CHO WT-CFTR cells (n = 81 cells). Mannitol to Nycodenz followed by Nycodenz to Mannitol solution exchange was performed before any stimulation (plateaus 1–2 and 3–4, respectively). After the forskolin application, another Mannitol to Nycodenz solution exchange was done (plateaus 5–6). Mean intracellular refractive index $\bar{n_{2}}$ and mean cellular thickness $\bar{e}$ were calculated from the OPD values obtained from the whole cell surface whereas OPD variation obtained from the nucleus region allowed us to follow cell responses to forskolin. Before the stimulation, no significant difference of $\bar{e}$ (1.923 ± 0.0576 μm vs 1.919 ± 0.0544 μm, p = 0.8322; Fig 4B) or $\bar{n_{2}}$ (1.3670 ± 0.0005 vs 1.3670 ± 0.0005, p = 0.7632; Fig 4C) were detected, suggesting that cells perturbation during the extracellular solution exchange is limited. However, we observed that application of forskolin induced a significant cellular thickness decrease of $\Delta \bar{e}$ = -0.216 ± 0.0438 μm (1.919 ± 0.0544 μm vs 1.704 ± 0.0555 μm, n = 81 cells, p < 0.001, Wilcoxon signed rank test, Fig 4B) and a significant intracellular refractive index increase of $\Delta \bar{n_{2}}$ = 0.006 ± 0.001 (1.3670 ± 0.0005 vs 1.3730 ± 0.001, n = 81 cells, p < 0.001, Wilcoxon signed rank test, Fig 4C). Using the cell thickness variations, the cell volume was estimated to decrease of ΔV_c_ = -124.7 ± 14.89 μm^3^ (790 ± 34.23 μm^3^ vs 665 ± 28.84 μm^3^, n = 81 cells, p < 0.001, Wilcoxon signed rank test).

In parallel, we also measured the Optical Volume Difference (OVD) [26] by integrating the OPD over the entire cell surface of CHO WT-CFTR cells to confirm that the OPD variation observed after forskolin application was not due to an increase in cellular dry mass (total cellular mass without water), which could also affect the intracellular refractive index. Forskolin addition significantly increased the OPD (24.83 ± 2.30 nm, n = 6) compared to the DMSO control (2.22 ± 0.76 nm, n = 9, p = 0.0022; S2 Fig) but no significant OVD variation was detected (forskolin: -0.95 ± 0.52 μm^3^ vs DMSO: 0.10 ± 0.65 μm^3^, p = 0.0649; S2 Fig).

Together, our observations suggest that the OPD increase recorded after the forskolin stimulation of CHO WT-CFTR cells is both related to a cellular volume decrease and an intracellular refractive index increase. This n_2_ variation could be due to an intracellular medium concentration following a water efflux as Jourdain et al concluded [22].

### Inhibition of the cAMP-dependent OPD increase by mercury chloride

**Fig 5:**
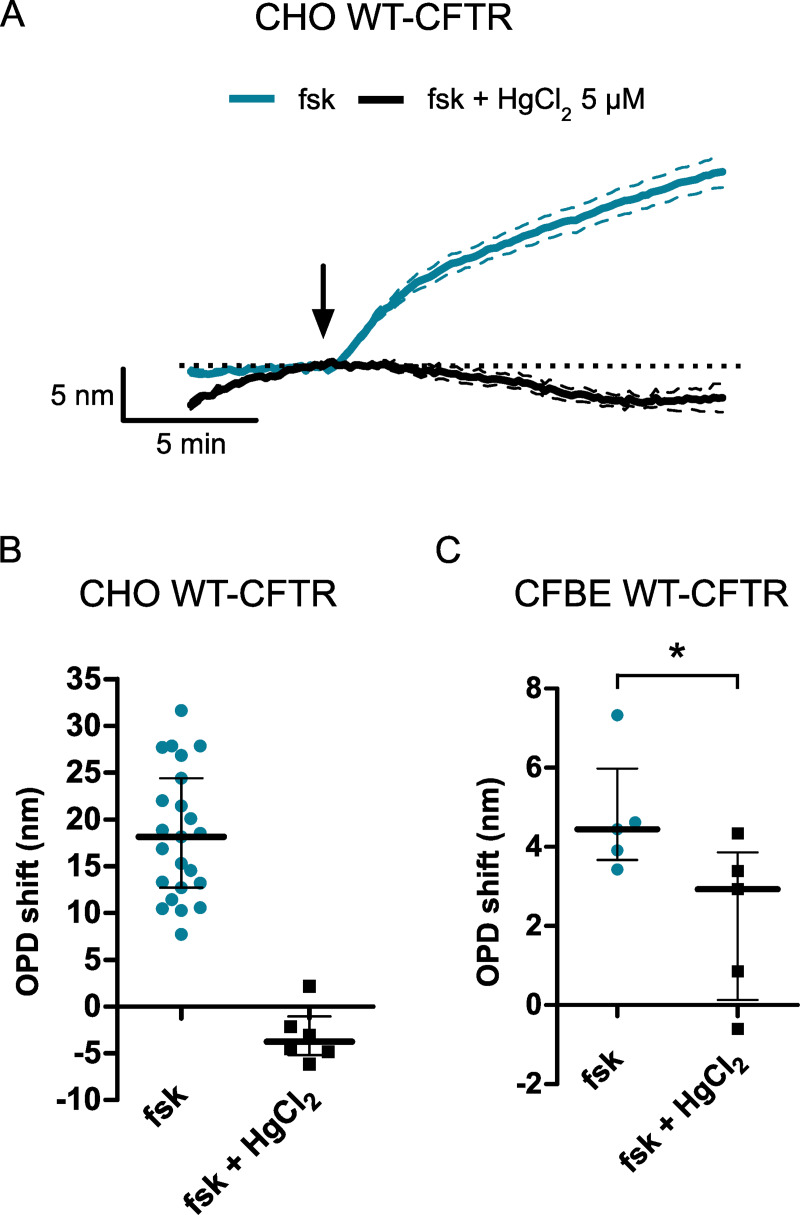
Inhibition of the forskolin-activated OPD increase by mercuric chloride. (A) Graphical representation of the OPD variation detected in CHO WT-CFTR cells in the absence or in the presence of HgCl_2_ (5 μM). (B, C) Quantification of the OPD variation measured after 20 min of recording in the absence or in the presence of HgCl_2_ (5 μM) for CHO WT-CFTR cells (B) (n = 23 and n = 6, respectively) and CFBE WT-CFTR cells (C) (n = 5 and n = 5, respectively) (* p < 0.05, *** p < 0.001, two-tailed Mann-Whitney test).

Previous studies using the DHM technique showed that cellular volume decrease and intracellular refractive index increase were consecutive to a transmembrane water efflux [19,21,22]. In the following experiments, we then incubated cells with mercuric chloride (HgCl_2_, 5 μM), 5 min before the forskolin stimulation to inhibit aquaporins (AQP), which are the major pathway for transmembrane water fluxes. Since the prolongated cell exposition to HgCl_2_ ended up being toxic, we measured the OPD shift after only 20 min of phase images recordings. An example of the effect of HgCl_2_ on the cAMP-dependent OPD increase time course is shown in the Fig 5A. Application of forskolin (10 μM) in the presence of HgCl_2_ failed to increase the OPD compared to forskolin stimulation alone in CHO WT-CFTR (p < 0.001; Fig 5A and 5B) and CFBE WT-CFTR cells (p = 0.0317; Fig 5C). These results suggest that the cAMP-dependent OPD increase activated in both CHO and CFBE WT-CFTR cells involves a water efflux dependent on an endogenous mercury-sensitive AQP.

**Fig 6:**
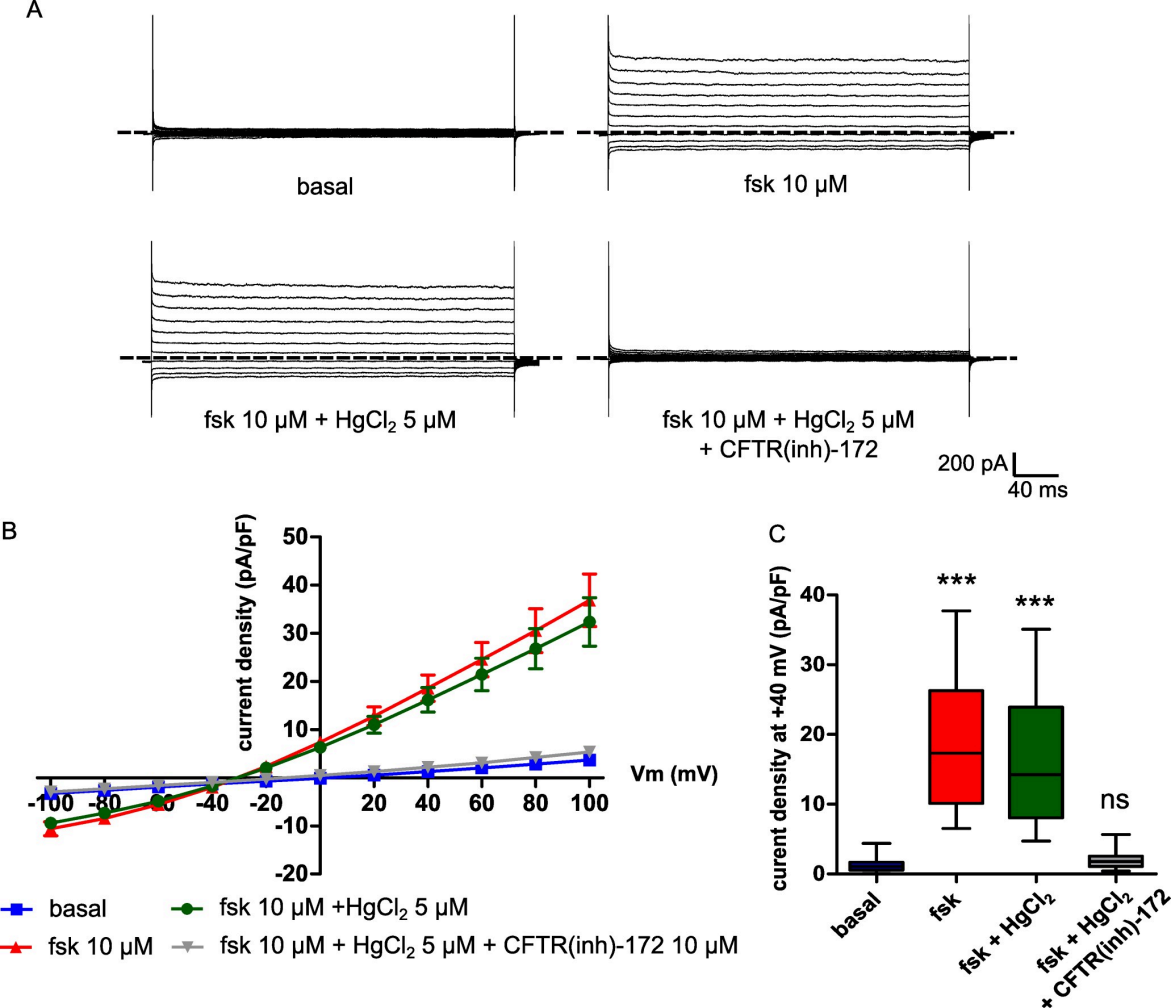
CFTR mediated currents in CHO WT-CFTR cells are not significantly impacted by mercuric chloride. (A) Representative whole-cell currents recorded in CHO WT-CFTR cells under control condition (basal), after forskolin perfusion (fsk, 10 μM), in the presence of HgCl_2_ (5 μM) or with HgCl_2_ (5 μM) and CFTR(inh)-172 (10 μM). (B) Corresponding mean current/voltage relationship and (C) Current density at +40 mV (pA/pF) for each condition described in (A). All conditions are statistically compared to the control condition (basal) (n = 13, *** p < 0.001, ns: non-significant, two-tailed Mann-Whitney test). Dotted lines indicate current baseline (0 pA).

**Fig 7:**
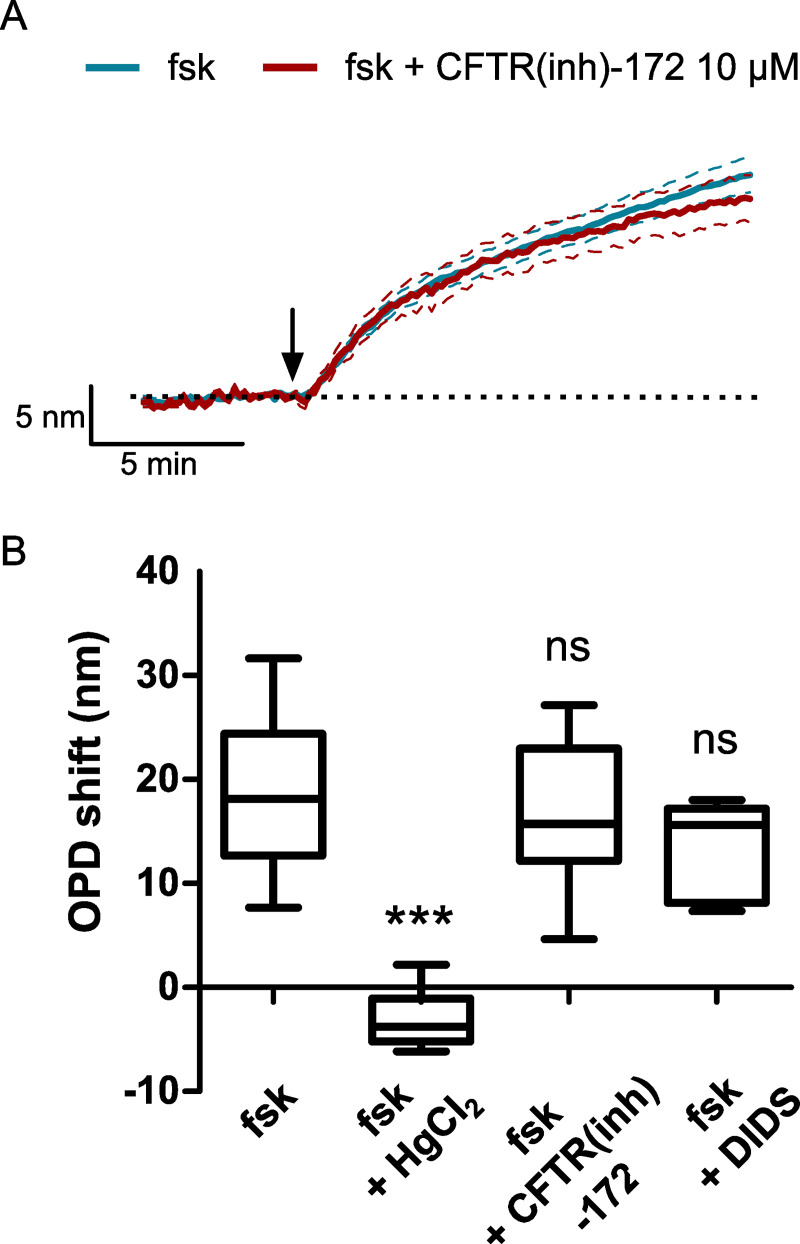
Effect of chloride currents inhibition on the forskolin-activated OPD increase in CHO WT-CFTR cells. (A) Time course of the OPD shift after forskolin application without (blue) or with (red) preincubation of CHO WT-CFTR with CFTR(inh)-172 (10 μM). (B) Quantification of the forskolin-activated OPD increase in the presence of HgCl_2_ (5 μM), CFTR(inh)-172 (10 μM) or DIDS (200 μM).

However, mercury could also disrupt membrane ion transports due to cellular toxic effects and inhibit osmotic driven water flux. To verify that mercury did not have an effect on CFTR cAMP-dependent activity, we also recorded cAMP-activated chloride currents in CHO WT-CFTR cells using the patch-clamp technique. The current density measured after forskolin stimulation (10 μM) was significantly higher than the control condition (basal) at +40 mV (basal: 1.27 ± 0.31 pA/pF; forskolin: 18.61 ± 2.71 pA/pF, n = 13, p < 0.001; Fig 6). Moreover, this forskolin-activated current was not significantly modified by HgCl_2_ (5 μM) (16.18 ± 2.59 pA/pF, n = 13, p = 0.5727; Fig 6) but was blocked by application of CFTR(inh)-172 (10 μM) (2.16 ± 0.42 pA/pF, n = 13, p < 0.001; Fig 6). We then recorded forskolin-stimulated OPD variations in CHO WT-CFTR cells in the presence of two chloride channels inhibitors, CFTR(inh)-172 (10 μM), a selective CFTR inhibitor [30], and 4,4’-diisothiocyanato-stilbene-2,2’-disulfonic acid (DIDS, 200 μM), a non-selective anion channel blocker which does no inhibit CFTR-dependent conductances. We observed that the OPD increase activated by 10 μM of forskolin (18.33 ± 1.43 nm, n = 23; Fig 7A and 7B) was not significantly modified in the presence of CFTR(inh)-172 (16.88 ± 1.96 nm, n = 12, p = 0.614; Fig 7A and 7B) or DIDS (13.56 ± 1.58 nm, n = 8, p = 0.0822; Fig 7B) compared to HgCl_2_ (-3.08 ± 1.20 nm, n = 6, p < 0.001; Fig 7B). Together, these results show that the inhibition of the forskolin-stimulated water efflux by mercury is dependent on an endogenous aquaporin and is not due to chloride transport disruption. Moreover, it suggests that the channel function of CFTR is not directly implicated in this water transport.

### Effect of the cystic fibrosis mutations G551D and F508del on the cAMP-activated OPD increase

**Fig 8:**
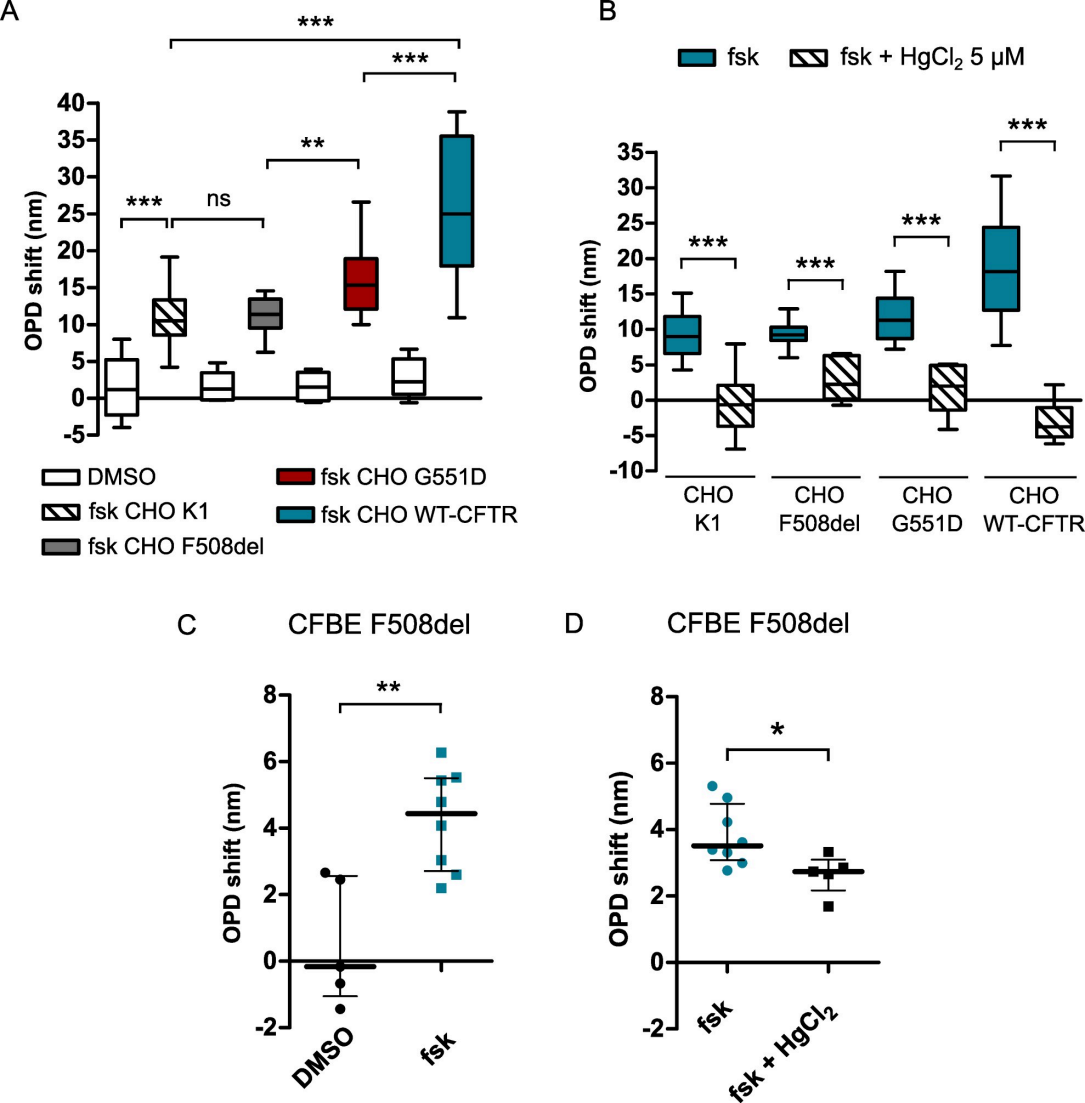
OPD variation stimulated by forskolin in cells lacking CFTR or expressing the G551D or F508del mutations and effect of mercuric chloride. (A) Quantification of the OPD variation in CHO K1 (n = 22), CHO F508del (n = 17), CHO G551D (n = 16) and CHO WT-CFTR (n = 23) cells after forskolin application or in control condition with DMSO. (B) Quantification of the OPD variation measured after 20 min of forskolin stimulation in the absence or in the presence of HgCl_2_ (5 μM) for CHO K1 (n = 22 and n = 6, respectively), CHO F508del (n = 16 and n = 17, respectively), CHO G551D (n = 13 and n = 6, respectively) and CHO WT-CFTR (n = 23 and n = 6, respectively). (C) Quantification of the OPD variation during forskolin stimulation (n = 8) compared to DMSO (0.1%, n = 5) in CFBE F508del cells. (D) Quantification of the OPD variation measured after 20 min of forskolin stimulation in the absence or in the presence of HgCl_2_ (5 μM) for CFBE F508del cells (n = 8 and n = 5, respectively) (* p < 0.05, ** p < 0.01, *** p < 0.001, ns: non-significant, two-tailed Mann-Whitney test).

To further investigate the implication of the CFTR channel in this cAMP-dependent water efflux, we recorded the OPD variation in CHO cells lacking CFTR expression (CHO K1 cells). Fig 8 shows that application of forskolin (10 μM) induced a significant OPD increase (10.77 ± 0.76 nm, n = 24; Fig 8A) compared to DMSO condition (1.41 ± 1.55 nm, n = 8, p < 0.001; Fig 8A) in CHO K1 cells. This result suggests that an endogenous water transport can be activated by an intracellular cAMP pathway even in the absence of CFTR. However, this response was significantly lower than the one observed in CHO WT-CFTR cells (24.89 ± 1.78 nm, n = 23, p < 0.001; Fig 8A), suggesting that the expression of CFTR induces an increase in cAMP-dependent water efflux.

In a second series of experiments, we reasoned that if the water transport is at least partially dependent on CFTR, then mutated channels with abnormal activity might impact the cAMP-dependent OPD response. To that end, we also recorded OPD variations in two other CHO cell lines expressing two disease-causing CFTR mutations, G551D (CHO G551D) and F508del (CHO F508del). The G551D mutated channel is present at the plasma membrane but has a markedly reduced channel activity [31] whereas the F508del mutated channel is sequestered in the endoplasmic reticulum and is thus barely expressed at the plasma membrane [10]. In CHO G551D cells, forskolin (10 μM) induced an increase of the OPD (16.12 ± 1.24 nm, n = 16; Fig 8A) which reached a value significantly higher than the response observed in both CHO K1 (10.77 ± 0.76 nm, n = 24, p = 0.0013; Fig 8A) and CHO F508del cells (11.24 ± 0.93 nm, n = 17, p = 0.0047; Fig 8A). Importantly, the OPD shift activated in CHO F508del cells was not significantly different from the response observed in CHO K1 cells (p = 0.5057). In a similar way to CHO WT-CFTR cells, a complete inhibition of the forskolin-dependent OPD increase by HgCl_2_ was observed in CHO K1, CHO G551D and CHO F508del cells (p < 0.001, Fig 8B).

Forskolin (10 μM) also activated a significant OPD increase (4.24 ± 0.53 nm, n = 8; Fig 8C) compared to DMSO (0.57 ± 0.84 nm, n = 5; p = 0.0109; Fig 8C) in CFBE cells expressing F508del-CFTR (CFBE F508del). This response was significantly reduced by mercury (p = 0.0186; Fig 8D). It was also noticed that the forskolin-stimulated OPD shift observed in CFBE F508del cells was not significantly different from the CFBE WT-CFTR cells response (p = 0.6485; see Fig 3A).

### Effect of the potentiator VX-770 on the cAMP-dependent water transport in G551D expressing cells

**Fig 9:**
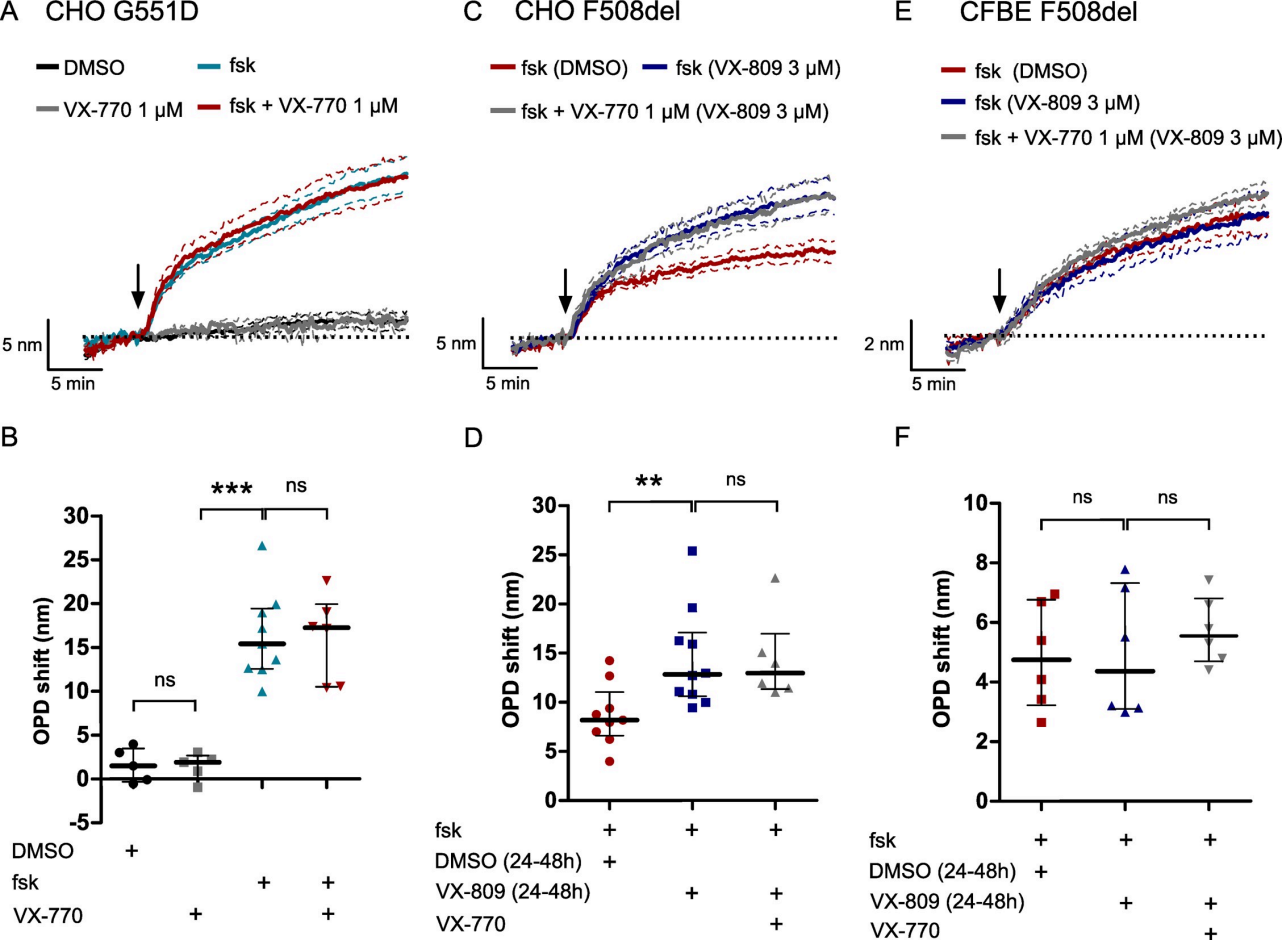
The forskolin-activated OPD increase is potentiated by the CFTR presence at the plasma membrane. (A) Time course and (B) comparison of the OPD variation in CHO G551D cells in control condition with DMSO (0.1%, n = 5), after VX-770 (1 μM, n = 5), forskolin (10 μM, n = 9) and forskolin + VX-770 addition (n = 6). (C) Time course and (D) comparison of the OPD variation obtained during forskolin stimulation in DMSO treated CHO F508del cells (0.01% v/v, n = 9) and after forskolin and forskolin + VX-770 addition in VX-809-corrected CHO F508del cells (3 μM, n = 10 and n = 6 respectively). (E) Time course and (F) comparison of the OPD variation obtained during forskolin stimulation in DMSO treated CFBE F508del cells (0.01%, n = 6) and after forskolin and forskolin + VX-770 addition in VX-809-corrected CFBE F508del cells (3 μM, n = 6 for both conditions). (** p < 0.01, *** p < 0.001, ns: non-significant, two-tailed Mann-Whitney test).

We then evaluated the effect of VX-770 (ivacaftor), a therapeutic potentiator used to improve the function of the G551D-CFTR channel activity by increasing its open state probability [15] with clinical benefit for CF patients [32]. Application of VX-770 (1 μM) alone did not induce a significant OPD shift (1.42 ± 0.69 nm, n = 5; Fig 9A) compared to the control condition with DMSO (1.56 ± 0.87, n = 5, p = 1.0000; Fig 9A) in CHO G551D cells. Addition of VX-770 (1 μM) in combination with forskolin (10 μM) triggered a significant OPD increase of 16.17 ± 1.97 nm (n = 6; p = 0.0010, Fig 9A and 9B), a value that was however not significantly different from the value obtained when forskolin was used without VX-770 (16.30 ± 1.68 nm, n = 9; p = 0.9546; Fig 9A and 9B), suggesting that increasing the G551D CFTR channel activity with VX-770 did not enhance the cAMP-activated water efflux.

### Effect of the corrector VX-809 on the cAMP-dependent water transport in F508del expressing cells

**Fig 10:**
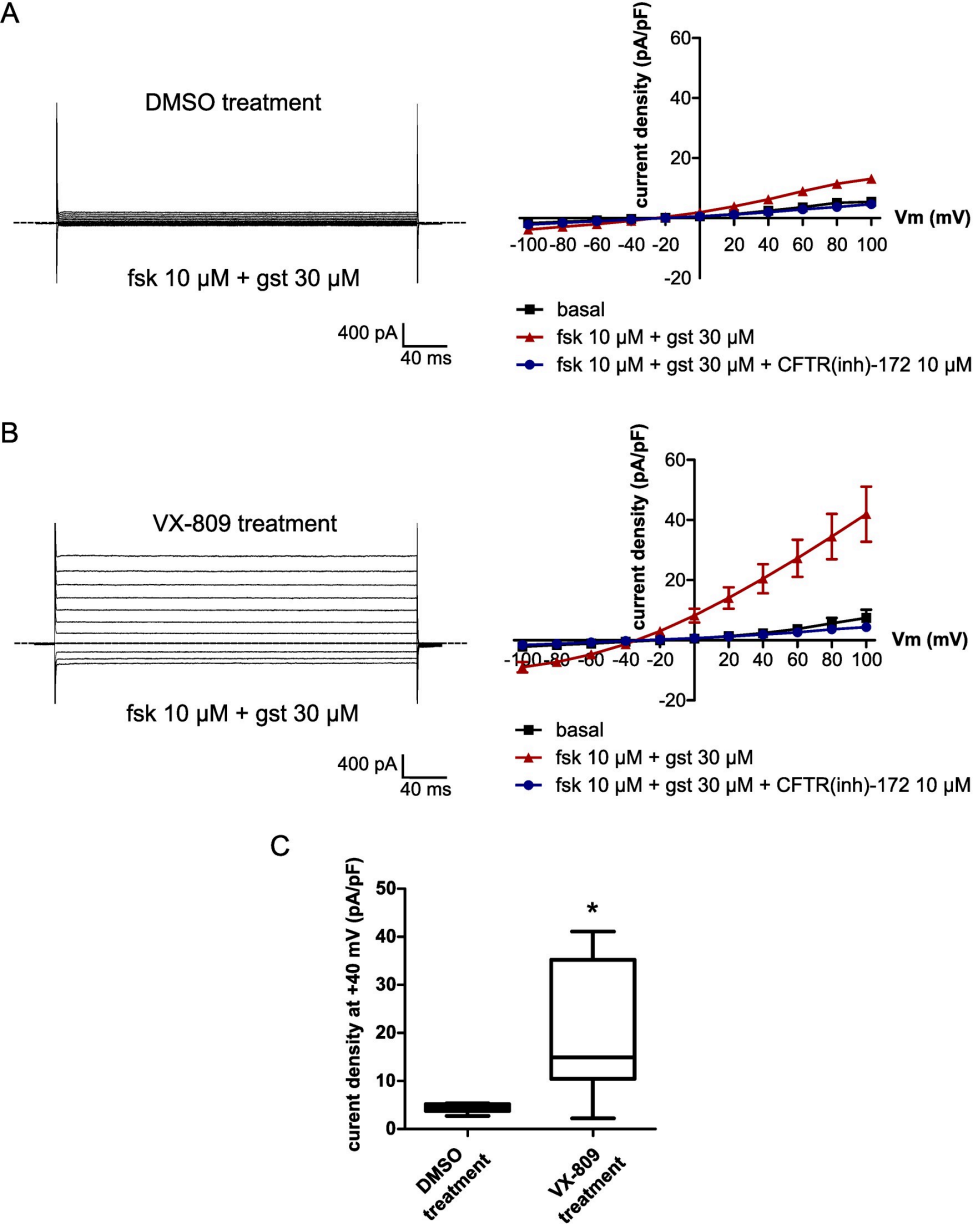
Restoration of CFTR function in CHO F508dels cells treated with the corrector VX-809. (A,B) (left) Representative whole-cell currents recorded in CHO F508del cells treated with (A) DMSO (0.1%, v/v, n = 6) or (B) VX-809 (3 μM, n = 7) after perfusion with forskolin (fsk, 10 μM) + genistein (gst, 30 μM) and (right) mean current/voltage relationship under control condition (basal) and after perfusion with forskolin (10 μM) + genistein (30 μM) without or with CFTR(inh)-172 (10 μM). (C) Current density at +40 mV (pA/pF) recorded in CHO F508del treated with DMSO (0.1%, v/v) or VX-809 (3 μM) after stimulation with forskolin (10 μM) + genistein (30 μM) (* p < 0.05,two-tailed Mann-Whitney test). Dotted lines indicate current baseline (0 pA). fsk: forskolin; gst: genistein.

Finally, we evaluated the effect of restoring F508del-CFTR protein localization at the plasma membrane on the forskolin-dependent water efflux of CHO F508del and CFBE F508del cells. To do so, we used the corrector drug VX-809 (lumacaftor) administrated to some CF patients [14]. Cells were incubated with 3 μM VX-809 or DMSO as vehicle for 24 to 48h prior to the phase imaging experiments. We observed that forskolin stimulated an OPD increase by 14.42 ± 1.58 nm (n = 10) in VX-809-corrected CHO F508del cells, a value significantly higher than the response obtained after DMSO treatment (8.72 ± 1.05 nm, n = 9, p = 0.0030; Fig 9C and 9D). Because CF patients with at least one F508del CFTR mutation are treated by Orkambi [33], the combination of VX-809 and VX-770, we also stimulated CHO F508del-VX-809 corrected cells with forskolin + VX-770. However, in this condition, addition of VX-770 did not further increase the cAMP-dependent water efflux (14.35 ± 1.78, n = 6, p = 0.8749; Fig 9C and 9D). We also did not observe an improvement of the OPD response stimulated by forskolin or forskolin + VX-770 after CFBE F508del cells correction with VX-809 compared to DMSO incubation (p = 0.9372 and p = 0.4848, respectively; Fig 9E and 9F). In parallel, correct F508del-CFTR protein reinsertion at the plasma membrane in CHO F508del cells was confirmed by patch-clamp experiments. The CFTR dependent current density measured at +40 mV after application of forskolin (10 μM) and the CFTR potentiator genistein (30 μM) was significantly higher in VX-809-corrected CHO F508del cells (19.10 ± 5.27 pA/pF, n = 7; Fig 10) than in DMSO-treated CHO F508del cells (4.41 ± 0.40 pA/pF, n = 6, p = 0.0266; Fig 10). Taken together, these results suggest that reinsertion of the mutated CFTR protein at the plasma membrane could enhance the cAMP-activated water efflux in F508del-CFTR expressing cells.

### AQP3 is the main aquaporin expressed in CHO and CFBE cells

**Fig 11:**
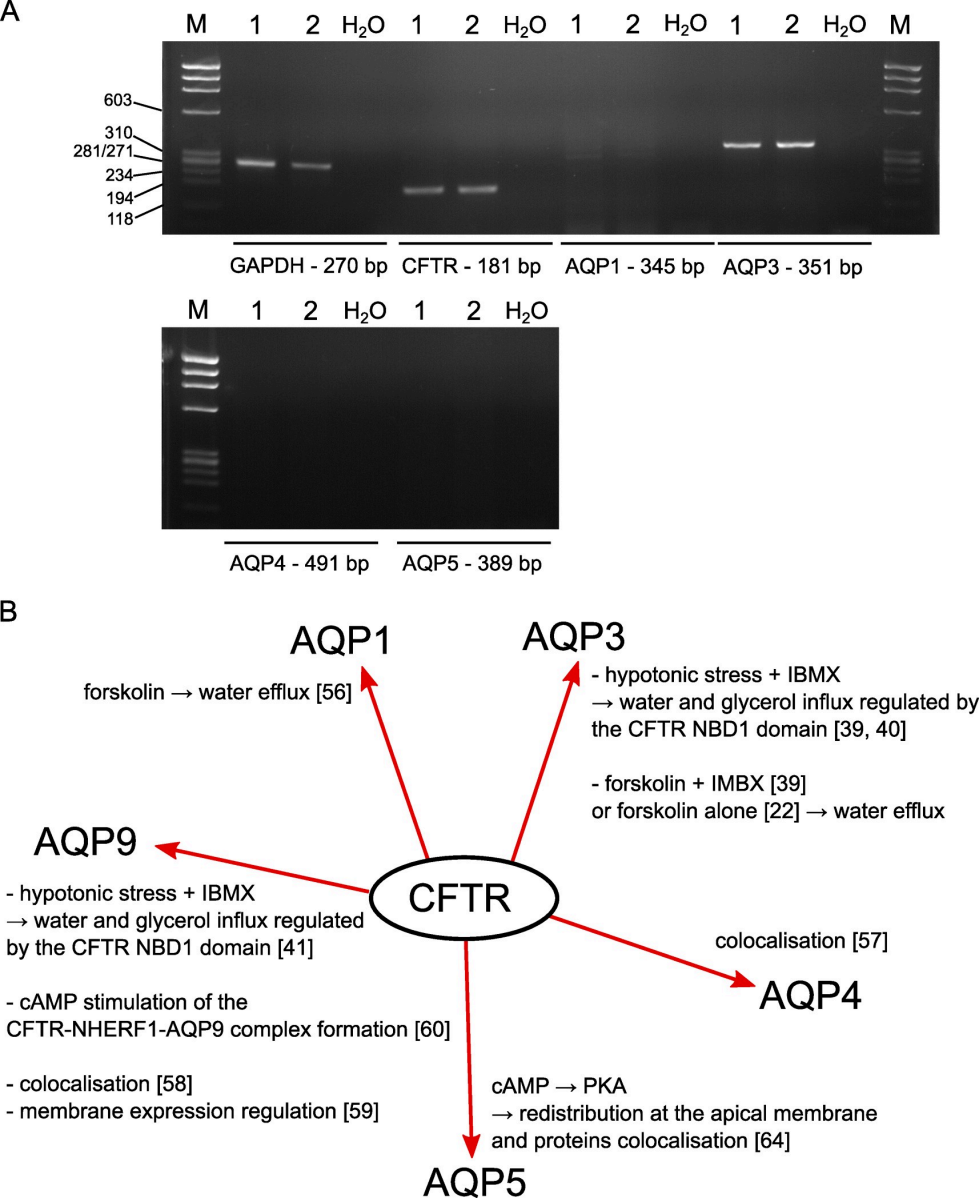
Detection of AQP mRNA transcripts in CFBE cells. (A) Analysis of CFTR, AQP1, AQP3, AQP4 and AQP5 mRNA transcripts expression in CFBE WT-CFTR (1) and CFBE F508del (2) cells by RT-PCR. M: marker, H_2_O: reaction without mRNA extract. (B) Summary of the reported aquaporins regulations by the CFTR channel.

To begin to understand what molecular entities are involved in the measured water efflux, we screened our cell models by RT-PCR to detect the mRNA transcripts of CFTR and several aquaporins. We have already shown that AQP3 is the major aquaporin expressed in CHO cells [22]. We then performed RT-PCR experiments in CFBE WT-CFTR and CFBE F508del cells to detect mRNA transcripts of AQP1, AQP3, AQP4 and AQP5, the main aquaporins expressed in the airway epithelia [34,35]. As shown in Fig 11A, together with CFTR mRNA, only AQP3 mRNA could be detected in CFBE cells. This observation suggests that AQP3 might participate to the transmembrane water flux in CFBE cells.

## Discussion

In our study, we first reported the ability to measure phase variations with the quantitative phase imaging technique based on quadriwave lateral shearing interferometry (QWLSI) during osmotic challenges applied on living cells. To do so, we used a PHASICS© module plugged onto a white-light conventional microscope which have been originally adapted to visualize cytoskeletal elements [25,36,37] or to obtain quantitative information such as cellular dry mass [26], without labeling. The QWLSI method appears to be sensitive enough to detect cell volume variations induced by a water influx or efflux after hypo or hyperosmotic challenges respectively.

We then showed that stimulation of the epithelial CFBE and non-epithelial CHO cells overexpressing WT-CFTR by forskolin increases OPD significantly. Previous studies using digital holographic microscopy (DHM) have shown that variations of optical responses (phase shift) result from intracellular concentration or dilution induced by water fluxes across cell membranes [19,21–23]. Here, our results suggest that this cAMP-dependent OPD variation was both due to a decrease in cellular volume and an increase in intracellular refractive index. Moreover, mercury completely inhibited this optical response suggesting that forskolin activates an AQP-dependent water efflux similarly to the observations of Jourdain et al [22].

In CHO cells, the cAMP-dependent OPD increase was potentiated by CFTR expression at the plasma membrane but did not seem to be dependent on the chloride transport function of the channel. Indeed, reinsertion of CFTR proteins into the plasma membrane by the corrector VX-809 in CHO F508del cells was accompanied by an increase of the forskolin-stimulated water-efflux. However, potentiation of CFTR function by VX-770 did not have a significant effect on the OPD responses measured in CHO G551D nor in CHO F508del-VX-809-corrected cells. We also did not observe a significant effect of the selective CFTR inhibitor, CFTR(inh)-172, on the forskolin-stimulated water efflux in CHO WT-CFTR cells.

Studying water transport in the genetic disease cystic fibrosis is relevant because, mutations of the CFTR gene disrupt chloride transport at the apical membrane of epithelial cells and lead to perturbed water secretion. Moreover, in a study using oocytes expressing CFTR, it was proposed that CFTR contains a cAMP-stimulated aqueous pore that can transport anions, water and small solutes [38]. Later, several studies have concluded that the CFTR channel activity positively regulates water transport mediated by aquaporins. More specifically, the cAMP stimulation of the CFTR chloride transport was shown to activate a water efflux through the two aquaglyceroporins AQP3 [39,40,22] and AQP9 [41]. Here, the optical responses measured in CHO G551D and CHO F508del cells suggest that the CFTR protein expression at the plasma membrane, but not directly its function, is necessary to increase the cAMP-dependent water efflux sensitive to HgCl_2_. Interestingly, we were able to detect a significant OPD increase after forskolin stimulation even in CHO cells lacking CFTR expression (CHO K1), suggesting the existence of an endogenous CFTR-independent mechanism of water flux regulation triggered by forskolin.

CFTR channel can also act as a protein regulator independently of its anion transport activity. For example, expression of the CFTR channel in HEK293 cells inhibited the volume-sensitive outwardly rectifying Cl^-^ channel (VSOR) current through the second nucleotide binding domain (NBD2) and independently of CFTR chloride transport [42]. SCL26A3 channel activation by cAMP in human colon is also dependent on CFTR expression but not on its transport activity [43]. The authors assumed that this regulation could involve PDZ-binding domains since such physical interaction has already been reported between CFTR and SLC26A3 [44]. A link between CFTR and AQP3 has been documented. Schreiber et al. concluded that CFTR can stimulate the AQP3-mediated water and glycerol transport in non-cystic fibrosis (non-CF) but not in cystic fibrosis (CF) airway epithelial cell lines [39]. The mechanism by which CFTR and AQP3 proteins physically interact or communicate through other mediators is nevertheless not yet determined. The first intracellular nucleotide binding domain (NBD1) of the transfected wild-type CFTR protein is said necessary to activate an endogenous AQP3 glycerol transport in oocytes after an increase of intracellular cAMP levels [40]. Interestingly, the authors still observed a significant increase in oocytes glycerol permeability when a CFTR fragment containing only the NBD1 was expressed, a part of the protein unable to form a functional chloride channel [45]. Similar observations were made concerning oocytes water permeability mediated by the transfected rat epidydimal AQP9 [41]. It was also demonstrated that lonidamine, a CFTR inhibitor binding to the NBD1 at high concentrations [46], reduced the potentiating effect of CFTR on AQP9 activity. It is then possible that CFTR regulates AQP3 activity through a direct interaction involving the NBD1. We observed that, in comparison with CHO K1 cells, forskolin can also stimulate a significantly higher water efflux in CHO G551D cells. However, G551D CFTR channels in CHO cells cannot mediate a cAMP-activated chloride current in the absence of a potentiator [47]. The water efflux amplitude was similar to the response detected in CHO F508del cells after VX-809 treatment but was still lower than in CHO CFTR-WT cells. Since the two mutations F508del and G551D are both located in the NBD1 [48], our observations also support a regulation of AQP3 by the CFTR NBD1. Of note, this CFTR domain, NBD1, has already been shown to be necessary for regulating several proteins such as the epithelial sodium channel (ENaC) [45,49] and the renal outer medullary potassium (ROMK) channels [50,51].

AQP-mediated water transport regulation by CFTR in CFBE cells might occur through a different mechanism. In the airways, AQP1, AQP3, AQP4 and AQP5 are the major aquaporins expressed [35]. AQP3 is localized at the basolateral membranes of the nasal epithelium and acinar cells forming submucosal glands but also in basal cells of the trachea [52]. Contrary to rodents, AQP3 can be detected at the apical membranes of proximal and terminal bronchioles of the human lung and was the only aquaporin identified in this region [34]. Because CFTR is expressed at the apical membrane of airway epithelial cells [53], we can therefore hypothesized that CFTR regulation of AQP3 requires a signaling pathway activation or other(s) partner(s), except for the bronchioles region, where the two proteins could have the possibility to physically interact. Interestingly recent observations proposed that AQPs participate to lung epithelial water transport upon durable changes in surface liquid volumes pointed to an underestimated role of AQPs in the lung [54]. Notably, AQP3 expression was transiently up-regulated several hours after apical volume expansion in cell models for the lung epithelium, NCI-H441 cell line-based epithelia and primary human tracheal epithelial cells [54]. Therefore, not only ion transport (ENaC and CFTR in particular) but also AQP are important to regulate water transport in the lung epithelia. In several organs and cells of different species, CFTR has been shown to interact with AQP1, AQP3, AQP4, AQP5 and AQP9 (summarized in the scheme Fig 8B). In rat and human pancreatic duct cells, both CFTR and AQP1 regulate the rate of pancreatic fluid secretion [55,56]. In rat Sertoli cells, AQP4 and AQP9 have been proposed to interact with CFTR [57,58]. In human syncytiotrophoblast from preeclamptic placentas AQP9 and CFTR colocalize in the apical membrane where CFTR may regulate AQP9 activity [59]. Similarly, CFTR and AQP9 co-localize in the apical membrane of principal cells of the epididymis and the vas deferens and NHERF1 and CFTR co-immunoprecipitate with AQP9 [60]. Thus, these studies confirmed that CFTR and various isoforms of AQP can interact directly or indirectly to control transmembrane water flux.

In human airway epithelial CFBE cells, we recorded a cAMP-regulated water efflux, the amplitude of which was however five times smaller than in recombinant CHO WT-CFTR cells. The mercury sensitivity of this cAMP-dependent water efflux and detection of AQP3 mRNA transcripts expression suggested an involvement of AQP3 in this response. Since the imaging technique used here is based on light deformation when passing through cells, it could be possible that when the CFBE cells are cultured as non-polarized monolayer, we can only detect limited variations of the optical signal because of a flatter shape than CHO cells. The difference in responses amplitude can also be due to the fact that CHO cells are commonly used to study transfected ion channels activities [61] whereas CFBE cells are more complex systems expressing numerous proteins such as the ENaC and TMEM16A channels [62,63] possibly activating regulatory pathways limiting large amplitude transmembrane water fluxes.

In conclusion, our study showed that the quantitative phase imaging based on quadriwave lateral shearing interferometry (QWLSI) could be a suitable technique to study transmembrane water fluxes in CFBE and CHO cells. We found that the CFTR channel expression at the plasma membrane, but not directly its channel function, can increase the cAMP-dependent water efflux sensitive to mercury, probably by regulating an endogenous AQP activity. Our study also shows abnormal responses in cells expressing two of the most common CF mutations, F508del and G551D. These observations could contribute to better understand the airway surface dehydration mechanisms in cystic fibrosis. Despite the fact that further studies will be needed to dissect the molecular mechanisms linking CFTR to AQP, our results thus provide additional evidence in favor of a role of CFTR in regulating water transport, which could help to identify potential means for therapeutic rehydration of cystic fibrosis airway epithelial cell surface.

## Supporting information

S1 Table Sequence primers for CFTR, AQP1, AQP3, AQP4, AQP5 and GAPDH genes detection by RT-PCR analysis. (TIF)

S1 Fig Representative phase images of CFBE and CHO WT-CFTR cells during osmotic challenges. Representative phase images of CFBE WT CFTR cells (A, B) and CHO WT CFTR cells (C, D) before (left), at the pic response (middle) and at the end (right) of a hypotonic challenge (A, C) or hypertonic challenge. (B, D). White ovals in the middle of the cells presented in the two inserts indicate the region of interest (ROI) where the OPD was recorded. Scale bar: 20 μm.
(TIF)

S2 Fig OPD and OVD variation after forskolin stimulation in CHO WT-CFTR cells. (A,B) Time course (left) and quantification (right) of maximal OPD (A) and OVD (B) variation after forskolin addition (arrow, 10 μM, blue) compared to control condition with DMSO (arrow, 0.1%, black) (n = 6 for each condition, ** p < 0.01, two-tailed Mann-Whitney test).
(TIF)

S1 Raw images (PDF)

## References

[1] Riordan JR. CFTR function and prospects for therapy. Annu Rev Biochem. 2008;77: 701–726. 10.1146/annurev.biochem.75.103004.142532 18304008

[2] Boucher RC. Muco-obstructive lung diseases. N Engl J Med. 2019;380: 1941–1953. 10.1056/NEJMra1813799 31091375

[3] Castellani C, Assael BM. Cystic fibrosis: a clinical view. Cell Mol Life Sci. 2017;74: 129–140. 10.1007/s00018-016-2393-9 27709245 PMC11107741

[4] Dean M. The human ATP-Binding Cassette (ABC) transporter superfamily. Genome Res. 2001;11: 1156–1166. 10.1101/gr.184901 11435397

[5] Riordan JR, Rommens JM, Kerem B-S, Alon N, Rozmahel R, Grzelczak Z, et al Identification of the Cystic Fibrosis Gene: cloning and characterization of complementary DNA. Science. 1989;245: 1066–1073. 10.1126/science.2475911 2475911

[6] Dulhanty AM, Riordan JR. Phosphorylation by cAMP-dependent protein kinase causes a conformational change in the R domain of the cystic fibrosis transmembrane conductance regulator. Biochemistry. 1994;33: 4072–4079. 10.1021/bi00179a036 7511414

[7] Liu F, Zhang Z, Levit A, Levring J, Touhara KK, Shoichet BK, et al Structural identification of a hotspot on CFTR for potentiation. Science. 2019;364: 1184–1188. 10.1126/science.aaw7611 31221859 PMC7184887

[8] Sheppard DN, Welsh MJ. Structure and Function of the CFTR Chloride Channel. Physiol Rev. 1999;79: S23–S45. 10.1152/physrev.1999.79.1.S23 9922375

[9] Tabcharani JA, Chang XB, Riordan JR, Hanrahan JW. Phosphorylation-regulated Cl- channel in CHO cells stably expressing the cystic fibrosis gene. Nature. 1991;352: 628–631. 10.1038/352628a0 1714039

[10] Cheng SH, Gregory RJ, Marshall J, Souza DW, White GA, D’Riordan F, et al Defective intracellular transport and processing of CFTR is the molecular basis of most cystic fibrosis. Cell. 1990;63: 827–834. 10.1016/0092-8674(90)90148-8 1699669

[11] Kopito RR. Biosynthesis and degradation of CFTR. Physiol Rev. 1999;79: S167–S173. 10.1152/physrev.1999.79.1.S167 9922380

[12] Dalemans W, Barbry P, Champigny G, Jallat S, Dott K, Dreyer D, et al Altered chloride ion channel kinetics associated with the delta F508 cystic fibrosis mutation. Nature. 1991;354: 526–528. 10.1038/354526a0 1722027

[13] Froux L, Billet A, Becq F. Modulating the cystic fibrosis transmembrane regulator and the development of new precision drugs. Expert Rev Precis Med Drug Dev. 2018;3: 357–370. 10.1080/23808993.2018.1547109

[14] Van Goor F, Hadida S, Grootenhuis PDJ, Burton B, Stack JH, Straley KS, et al Correction of the F508del-CFTR protein processing defect in vitro by the investigational drug VX-809. Proc Natl Acad Sci U S A. 2011;108: 18843–18848. 10.1073/pnas.1105787108 21976485 PMC3219147

[15] Van Goor F, Hadida S, Grootenhuis PDJ, Burton B, Cao D, Neuberger T, et al Rescue of CF airway epithelial cell function in vitro by a CFTR potentiator, VX-770. Proc Natl Acad Sci U S A. 2009;106: 18825–18830. 10.1073/pnas.0904709106 19846789 PMC2773991

[16] Saint-Criq V, Gray MA. Role of CFTR in epithelial physiology. Cellular and Molecular Life Sciences. 2017;74: 93–115. 10.1007/s00018-016-2391-y 27714410 PMC5209439

[17] Mall MA. Unplugging Mucus in Cystic Fibrosis and Chronic Obstructive Pulmonary Disease. Ann Am Thorac Soc. 2016; S177–185. 10.1513/AnnalsATS.201509-641KV 27115954

[18] De Rose V, Molloy K, Gohy S, Pilette C, Greene CM. Airway epithelium dysfunction in cystic fibrosis and COPD. Mediators Inflamm. 2018;2018: 1–20. 10.1155/2018/1309746 29849481 PMC5911336

[19] Jourdain P, Boss D, Rappaz B, Moratal C, Hernandez M-C, Depeursinge C, et al Simultaneous optical recording in multiple cells by digital holographic microscopy of chloride current associated to activation of the ligand-gated chloride channel GABAA receptor. PLoS ONE. 2012;7: e51041 10.1371/journal.pone.0051041 23236427 PMC3517575

[20] Rappaz B, Marquet P, Cuche E, Emery Y, Depeursinge C, Magistretti PJ. Measurement of the integral refractive index and dynamic cell morphometry of living cells with digital holographic microscopy. Opt Express. 2005;13: 9361 10.1364/OPEX.13.009361 19503137

[21] Jourdain P, Pavillon N, Moratal C, Boss D, Rappaz B, Depeursinge C, et al Determination of transmembrane water fluxes in neurons elicited by glutamate ionotropic receptors and by the cotransporters KCC2 and NKCC1: a digital holographic microscopy study. J Neurosci. 2011;31: 11846–11854. 10.1523/JNEUROSCI.0286-11.2011 21849545 PMC6623187

[22] Jourdain P, Becq F, Lengacher S, Boinot C, Magistretti PJ, Marquet P. The human CFTR protein expressed in CHO cells activates aquaporin-3 in a cAMP-dependent pathway: study by digital holographic microscopy. J Cell Sci. 2014;127: 546–556. 10.1242/jcs.133629 24338365

[23] Boss D, Kühn J, Jourdain P, Depeursinge C, Magistretti PJ, Marquet P. Measurement of absolute cell volume, osmotic membrane water permeability, and refractive index of transmembrane water and solute flux by digital holographic microscopy. J Biomed Opt. 2013;18: 036007 10.1117/1.JBO.18.3.036007 23487181

[24] Pavillon N, Benke A, Boss D, Moratal C, Kühn J, Jourdain P, et al Cell morphology and intracellular ionic homeostasis explored with a multimodal approach combining epifluorescence and digital holographic microscopy. J Biophotonics. 2010;3: 432–436. 10.1002/jbio.201000018 20306502

[25] Bon P, Maucort G, Wattellier B, Monneret S. Quadriwave lateral shearing interferometry for quantitative phase microscopy of living cells. Opt Express. 2009;17: 13080 10.1364/OE.17.013080 19654713

[26] Aknoun S, Savatier J, Bon P, Galland F, Abdeladim L, Wattellier B, et al Living cell dry mass measurement using quantitative phase imaging with quadriwave lateral shearing interferometry: an accuracy and sensitivity discussion. J Biomed Opt. 2015;20: 126009 10.1117/1.JBO.20.12.126009 26720876

[27] Okada Y. Ion channels and transporters involved in cell volume regulation and sensor mechanisms. Cell Biochem Biophys. 2004;41: 233–258. 10.1385/CBB:41:2:233 15475611

[28] Hoffmann EK, Lambert IH, Pedersen SF. Physiology of Cell Volume Regulation in Vertebrates. Physiol Rev. 2009;89: 193–277. 10.1152/physrev.00037.2007 19126758

[29] Jentsch TJ. VRACs and other ion channels and transporters in the regulation of cell volume and beyond. Nat Rev Mol Cell Biol. 2016;17: 293–307. 10.1038/nrm.2016.29 27033257

[30] Ma T, Thiagarajah JR, Yang H, Sonawane ND, Folli C, Galietta LJV, et al Thiazolidinone CFTR inhibitor identified by high-throughput screening blocks cholera toxin–induced intestinal fluid secretion. J Clin Invest. 2002;110: 1651–1658. 10.1172/JCI16112 12464670 PMC151633

[31] Drumm ML, Wilkinson DJ, Smit LS, Worrell RT, Strong TV, Frizzell RA, et al Chloride conductance expressed by delta F508 and other mutant CFTRs in Xenopus oocytes. Science. 1991;254: 1797–1799. 10.1126/science.1722350 1722350

[32] Accurso FJ, Rowe SM, Clancy JP, Boyle MP, Dunitz JM, Durie PR, et al Effect of VX-770 in persons with cystic fibrosis and the G551D-CFTR mutation. N Engl J Med. 2010;363: 1991–2003. 10.1056/NEJMoa0909825 21083385 PMC3148255

[33] Wainwright CE, Elborn JS, Ramsey BW, Marigowda G, Huang X, Cipolli M, et al Lumacaftor-Ivacaftor in Patients with Cystic Fibrosis Homozygous for Phe508del CFTR. N Engl J Med. 2015;373: 220–231. 10.1056/NEJMoa1409547 25981758 PMC4764353

[34] Kreda SM, Gynn MC, Fenstermacher DA, Boucher RC, Gabriel SE. Expression and Localization of Epithelial Aquaporins in the Adult Human Lung. Am J Respir Cell Mol Biol. 2001;24: 224–234. 10.1165/ajrcmb.24.3.4367 11245621

[35] Verkman AS. Role of aquaporins in lung liquid physiology. Respir Physiol Neurobiol. 2007;159: 324–330. 10.1016/j.resp.2007.02.012 17369110 PMC3315286

[36] Bon P, Savatier J, Merlin M, Wattellier B, Monneret S. Optical detection and measurement of living cell morphometric features with single-shot quantitative phase microscopy. J Biomed Opt. 2012;17: 0760041 10.1117/1.JBO.17.7.076004 22894487

[37] Bon P, Lécart S, Fort E, Lévêque-Fort S. Fast label-free cytoskeletal network imaging in living mammalian cells. Biophys J. 2014;106: 1588–1595. 10.1016/j.bpj.2014.02.023 24739158 PMC4008798

[38] Hasegawa H, Skach W, Baker O, Calayag M, Lingappa V, Verkman A. A multifunctional aqueous channel formed by CFTR. Science. 1992;258: 1477–1479. 10.1126/science.1279809 1279809

[39] Schreiber R, Nitschke R, Greger R, Kunzelmann K. The cystic fibrosis transmembrane conductance regulator activates aquaporin 3 in airway epithelial cells. J Biol Chem. 1999;274: 11811–11816. 10.1074/jbc.274.17.11811 10206998

[40] Schreiber R, Pavenstädt H, Greger R, Kunzelmann K. Aquaporin 3 cloned from Xenopus laevis is regulated by the cystic fibrosis transmembrane conductance regulator. FEBS Lett. 2000;475: 291–295. 10.1016/s0014-5793(00)01689-6 10869574

[41] Cheung KH, Leung CT, Leung GPH, Wong PYD. Synergistic effects of cystic fibrosis transmembrane conductance regulator and aquaporin-9 in the rat epididymis. Biol Reprod. 2003;68: 1505–1510. 10.1095/biolreprod.102.010017 12606488

[42] Ando-Akatsuka Y, Abdullaev I, Lee E, Okada Y, Sabirov R. Down-regulation of volume-sensitive Cl—channels by CFTR is mediated by the second nucleotide-binding domain. Pflugers Arch. 2002;445: 177–186. 10.1007/s00424-002-0920-z 12457238

[43] Tse C-M, Yin J, Singh V, Sarker R, Lin R, Verkman AS, et al cAMP stimulates SLC26A3 activity in human colon by a CFTR-dependent mechanism that does not require CFTR activity. Cell Mol Gastroenterol Hepatol. 2019;7: 641–653. 10.1016/j.jcmgh.2019.01.002 30659943 PMC6438990

[44] Ko S, Zeng W, Dorwart M, Luo X, Kim KH, Millen L, et al Gating of CFTR by the STAS domain of SLC26 transporters. Nat Cell Biol. 2004;6: 343–350. 10.1038/ncb1115 15048129 PMC3943213

[45] Schreiber R, Hopf A, Mall M, Greger R, Kunzelmann K. The first-nucleotide binding domain of the cystic-fibrosis transmembrane conductance regulator is important for inhibition of the epithelial Na+ channel. Proc Natl Acad Sci U S A. 1999;96: 5310–5315. 10.1073/pnas.96.9.5310 10220462 PMC21860

[46] Gong XD, Linsdell P, Cheung KH, Leung GPH, Wong PYD. Indazole inhibition of Cystic Fibrosis Transmembrane Conductance Regulator Cl− channels in rat epididymal epithelial cells. Biol Reprod. 2002;67: 1888–1896. 10.1095/biolreprod.102.007450 12444067

[47] Dérand R, Bulteau-Pignoux L, Mettey Y, Zegarra-Moran O, Howell LD, Randak C, et al Activation of G551D CFTR channel with MPB-91: regulation by ATPase activity and phosphorylation. Am J Physiol Cell Physiol. 2001;281: C1657–C1666. 10.1152/ajpcell.2001.281.5.C1657 11600430

[48] Logan J, Hiestand D, Daram P, Huang Z, Muccio DD, Hartman J, et al Cystic fibrosis transmembrane conductance regulator mutations that disrupt nucleotide binding. J Clin Invest. 1994;94: 228–236. 10.1172/JCI117311 7518829 PMC296301

[49] Kunzelmann K, Kiser GL, Schreiber R, Riordan JR. Inhibition of epithelial Na+ currents by intracellular domains of the cystic fibrosis transmembrane conductance regulator. FEBS Lett. 1997;400: 341–344. 10.1016/s0014-5793(96)01414-7 9009227

[50] McNicholas CM, Nason MW, Guggino WB, Schwiebert EM, Hebert SC, Giebisch G, et al A functional CFTR-NBF1 is required for ROMK2-CFTR interaction. Am J Physiol Renal Physiol. 1997;273: F843–F848. 10.1152/ajprenal.1997.273.5.F843 9374850

[51] Ruknudin A, Schulze DH, Sullivan SK, Lederer WJ, Welling PA. Novel subunit composition of a renal epithelial K_ATP_ channel. J Biol Chem. 1998;273: 14165–14171. 10.1074/jbc.273.23.14165 9603917

[52] Nielsen S, King LS, Christensen BM, Agre P. Aquaporins in complex tissues. II. Subcellular distribution in respiratory and glandular tissues of rat. Am J Physiol Cell Physiol. 1997;273: C1549–C1561. 10.1152/ajpcell.1997.273.5.C1549 9374640

[53] Engelhardt JF, Zepeda M, Cohn JA, Yankaskas JR, Wilson JM. Expression of the cystic fibrosis gene in adult human lung. J Clin Invest. 1994;93: 737–749. 10.1172/JCI117028 7509347 PMC293915

[54] Schmidt H, Michel C, Braubach P, Fauler M, Neubauer D, Thompson KE, et al Water permeability adjusts resorption in lung epithelia to increased apical surface liquid volumes. Am J Respir Cell Mol Biol. 2017;56: 372–382. 10.1165/rcmb.2016-0161OC 27814452

[55] Ko SBH, Yamamoto A, Azuma S, Song H, Kamimura K, Nakakuki M, et al Effects of CFTR gene silencing by siRNA or the luminal application of a CFTR activator on fluid secretion from guinea-pig pancreatic duct cells. Biochem Biophys Res Commun. 2011;410: 904–909. 10.1016/j.bbrc.2011.06.093 21708133

[56] Venglovecz V, Pallagi P, Kemény LV, Balázs A, Balla Z, Becskeházi E, et al The importance of aquaporin 1 in pancreatitis and its relation to the CFTR Cl- channel. Front Physiol. 2018;9 10.3389/fphys.2018.00854 30050452 PMC6052342

[57] Jesus TT, Bernardino RL, Martins AD, Sá R, Sousa M, Alves MG, et al Aquaporin-4 as a molecular partner of cystic fibrosis transmembrane conductance regulator in rat Sertoli cells. Biochem Biophys Res Commun. 2014;446: 1017–1021. 10.1016/j.bbrc.2014.03.046 24657265

[58] Jesus TT, Bernardino RL, Martins AD, Sá R, Sousa M, Alves MG, et al Aquaporin-9 is expressed in rat Sertoli cells and interacts with the cystic fibrosis transmembrane conductance regulator: Aquaporin-9 and CFTR In Sertoli Cells. IUBMB Life. 2014;66: 639–644. 10.1002/iub.1312 25270793

[59] Castro-Parodi M, Levi L, Dietrich V, Zotta E, Damiano AE. CFTR may modulate AQP9 functionality in preeclamptic placentas. Placenta. 2009;30: 642–648. 10.1016/j.placenta.2009.04.012 19481256

[60] Pietrement C, Da Silva N, Silberstein C, James M, Marsolais M, Van Hoek A, et al Role of NHERF1, Cystic Fibrosis Transmembrane Conductance Regulator, and cAMP in the regulation of aquaporin 9. J Biol Chem. 2008;283: 2986–2996. 10.1074/jbc.M704678200 18055461

[61] Gamper N, Stockand JD, Shapiro MS. The use of Chinese hamster ovary (CHO) cells in the study of ion channels. J Pharmacol Toxicol Methods. 2005;51: 177–185. 10.1016/j.vascn.2004.08.008 15862463

[62] Varelogianni G, Hussain R, Strid H, Oliynyk I, Roomans GM, Johannesson M. The effect of ambroxol on chloride transport, CFTR and ENaC in cystic fibrosis airway epithelial cells. Cell Biol Int. 2013;37: 1149–1156. 10.1002/cbin.10146 23765701

[63] Benedetto R, Ousingsawat J, Wanitchakool P, Zhang Y, Holtzman MJ, Amaral M, et al Epithelial Chloride Transport by CFTR Requires TMEM16A. Sci Rep. 2017;7 10.1038/s41598-017-10910-0 28963502 PMC5622110

