# Supplementary figures and images for: Quantitative phase imaging to study transmembrane water fluxes regulated by CFTR and AQP3 in living human airway epithelial CFBE cells and CHO cells
Source: PLoS One. 2020 May 29;15(5):e0233439. doi: 10.1371/journal.pone.0233439 (PMC7259668; doi:10.1371/journal.pone.0233439)

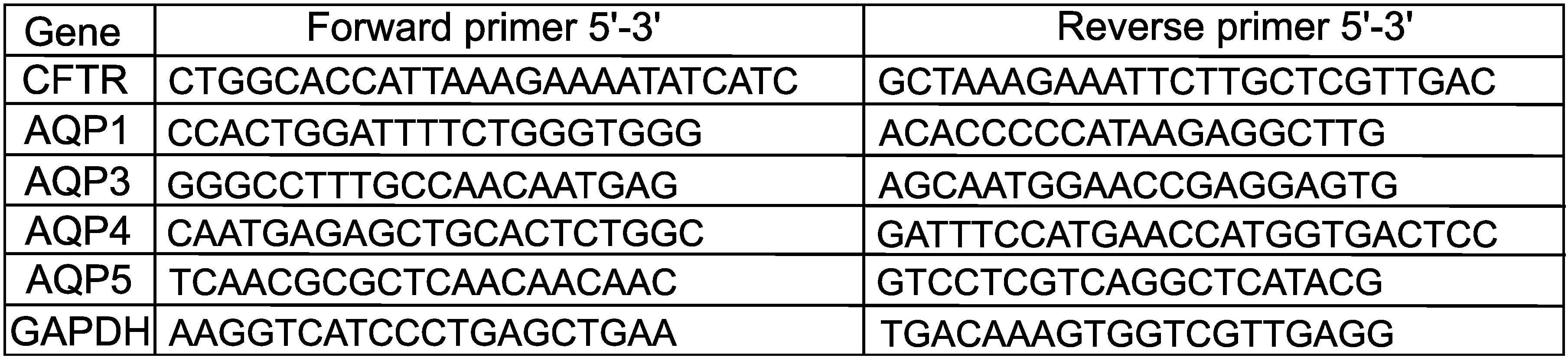

Supplement: S1 Table — (TIF) [file pone.0233439.s001.tif]

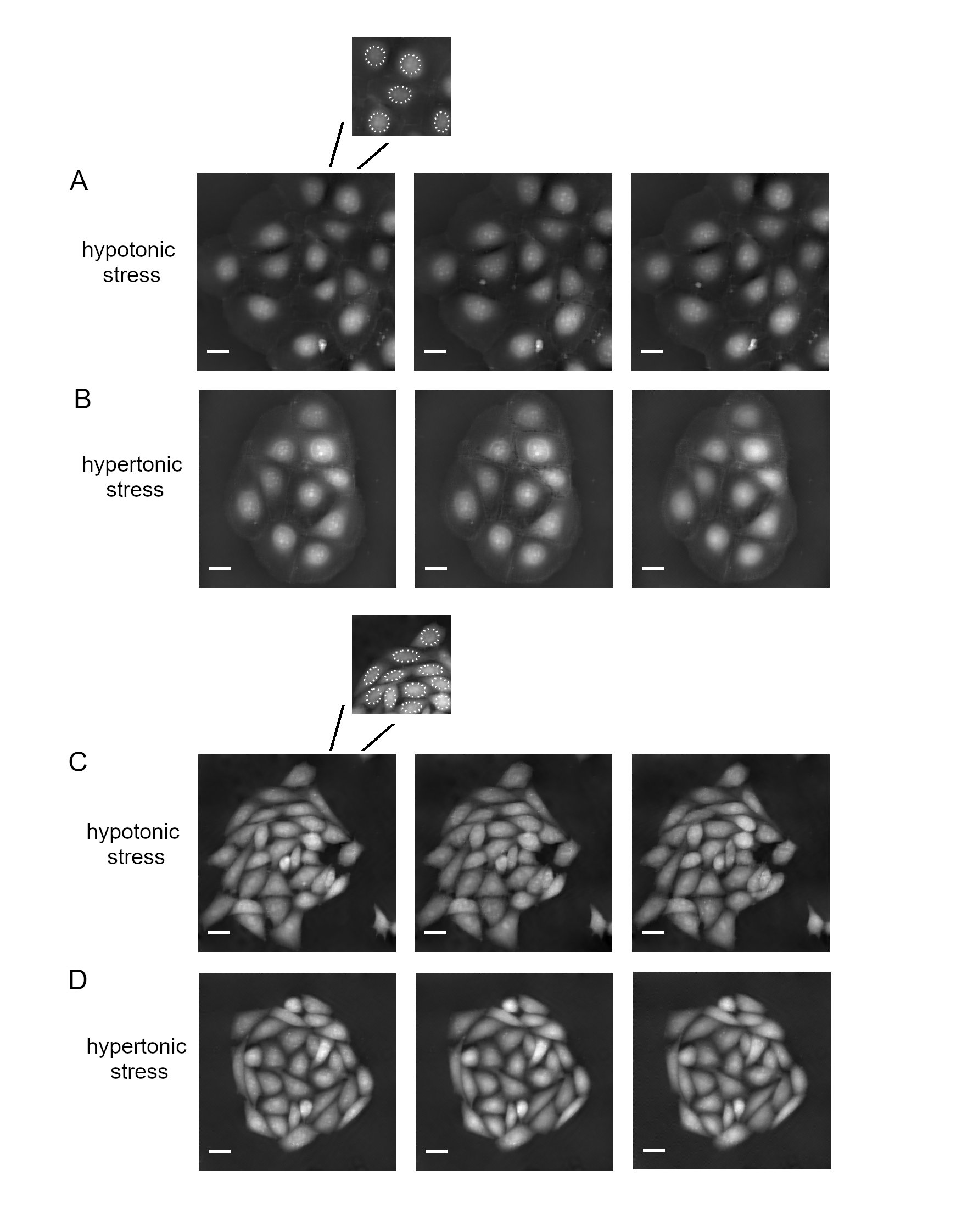

Supplement: S1 Fig — Representative phase images of CFBE WT CFTR cells (A, B) and CHO WT CFTR cells (C, D) before (left), at the pic response (middle) and at the end (right) of a hypotonic challenge (A, C) or hypertonic challenge. (B, D). White ovals in the middle of the cells presented in the two inserts indicate the region of interest (ROI) where the OPD was recorded. Scale bar: 20 μm. (TIF) [file pone.0233439.s002.tif]

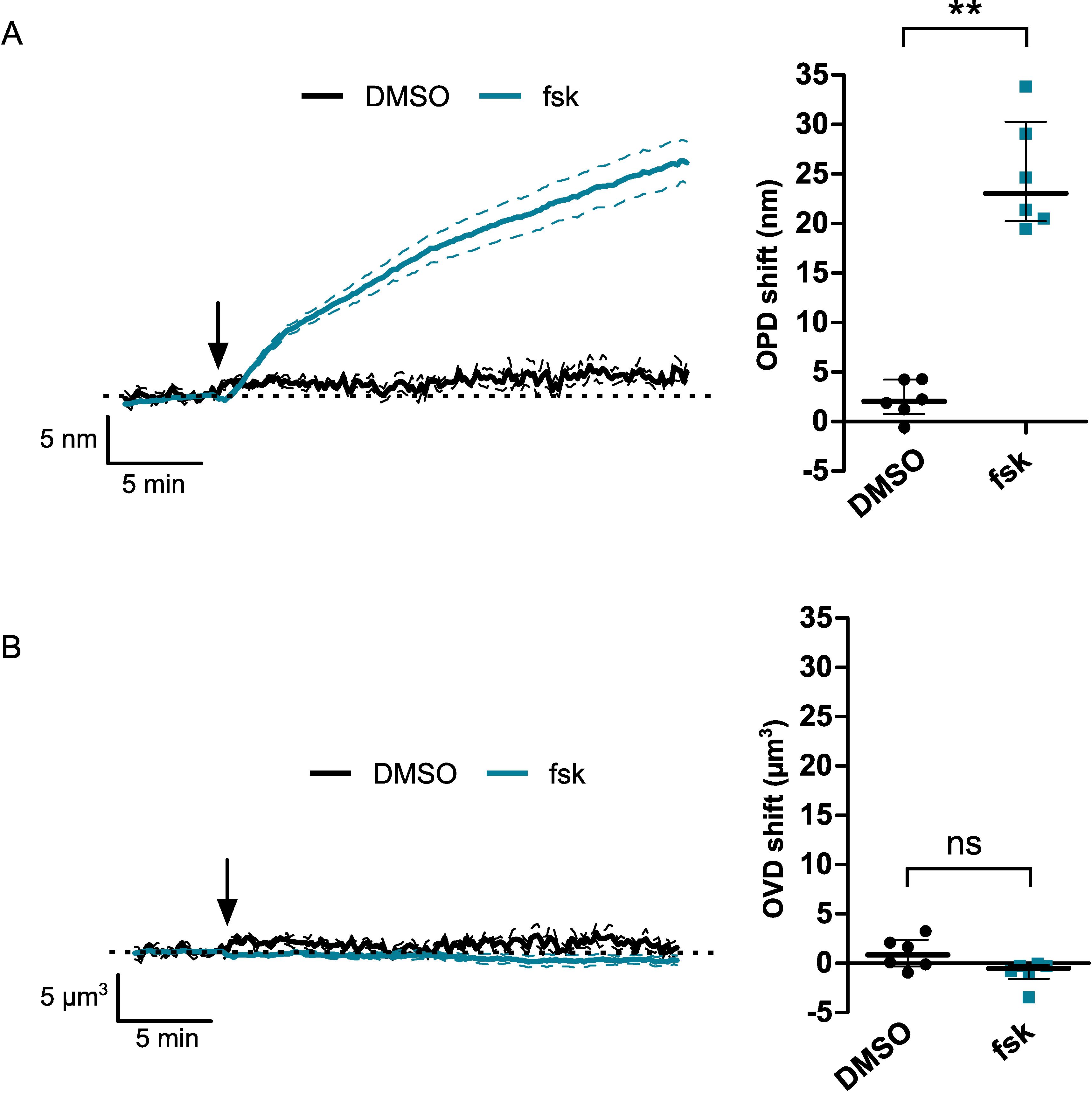

Supplement: S2 Fig — (A,B) Time course (left) and quantification (right) of maximal OPD (A) and OVD (B) variation after forskolin addition (arrow, 10 μM, blue) compared to control condition with DMSO (arrow, 0.1%, black) (n = 6 for each condition, ** p < 0.01, two-tailed Mann-Whitney test). (TIF) [file pone.0233439.s003.tif]

Figure 8A  
upper gel

1353  
1078  
872  
603  
310  
281/271  
234  
194

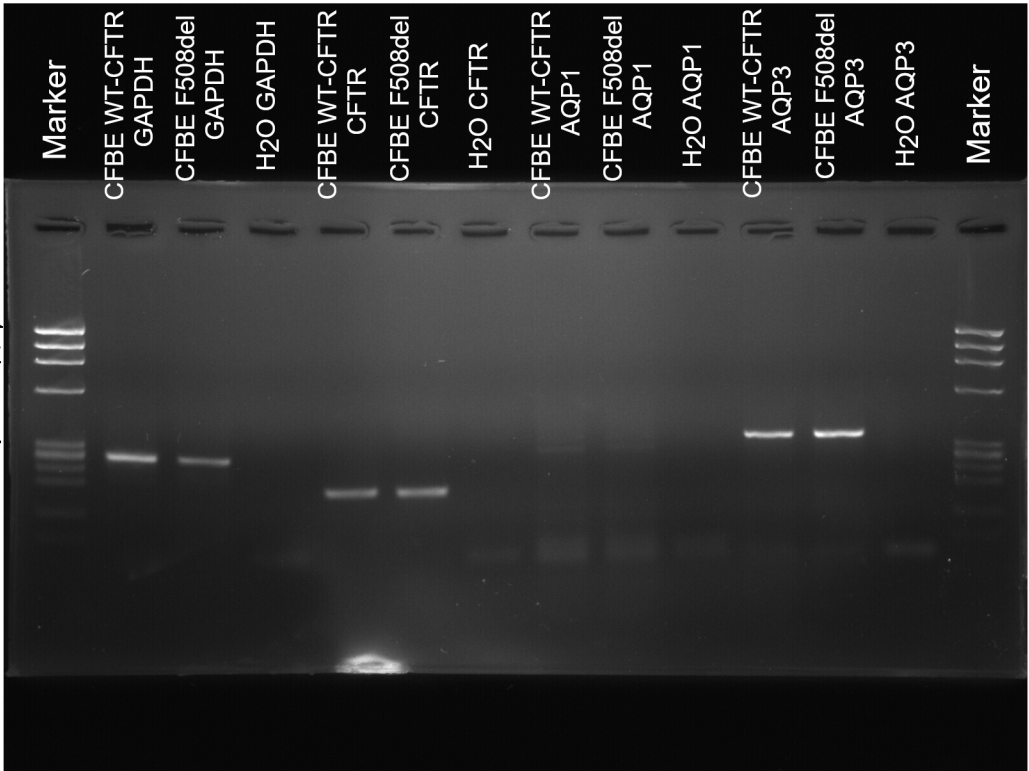

X X X X X X

Figure 8A  
lower gel

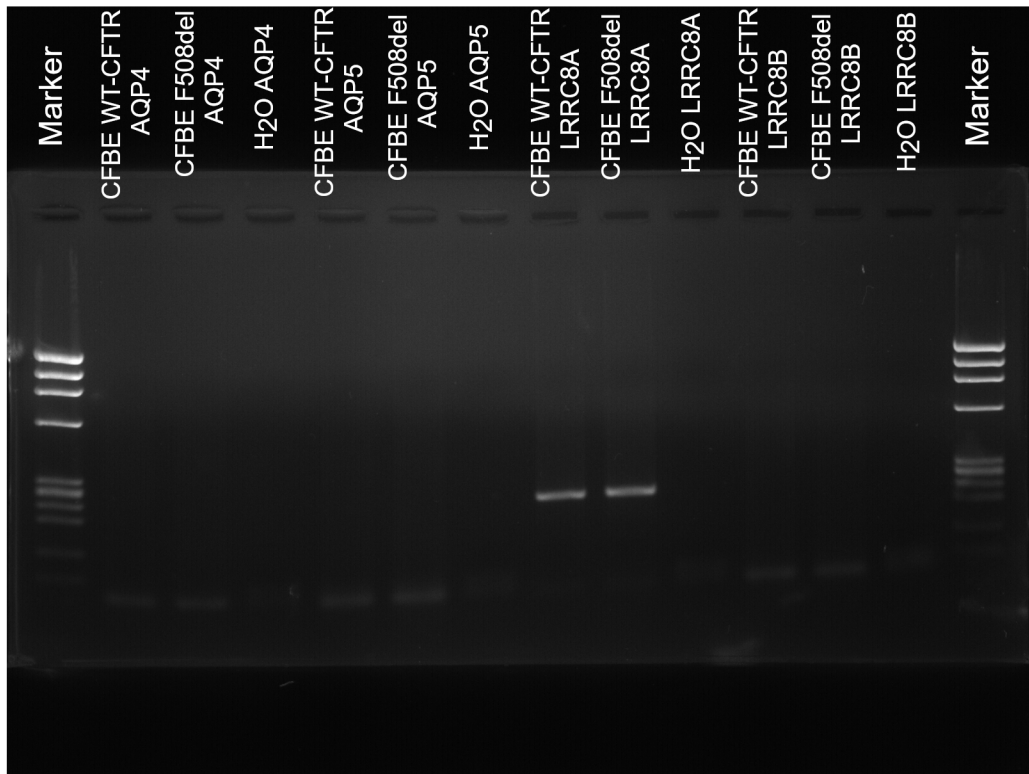

Supplement: S1 Raw images — (PDF) [file pone.0233439.s004.pdf]
